# Simulating Data From Marginal Structural Models for a Survival Time Outcome

**DOI:** 10.1002/bimj.70010

**Published:** 2024-11-23

**Authors:** Shaun R. Seaman, Ruth H. Keogh

**Affiliations:** ^1^ MRC Biostatistics Unit University of Cambridge Cambridge UK; ^2^ Department of Medical Statistics London School of Hygiene and Tropical Medicine London UK

**Keywords:** bootstrap, causal inference, compatible models, congenial models, continuous‐time marginal structural model, sandwich estimator, simulation studies, survival analysis, time‐dependent confounding

## Abstract

Marginal structural models (MSMs) are often used to estimate causal effects of treatments on survival time outcomes from observational data when time‐dependent confounding may be present. They can be fitted using, for example, inverse probability of treatment weighting (IPTW). It is important to evaluate the performance of statistical methods in different scenarios, and simulation studies are a key tool for such evaluations. In such simulation studies, it is common to generate data in such a way that the model of interest is correctly specified, but this is not always straightforward when the model of interest is for potential outcomes, as is an MSM. Methods have been proposed for simulating from MSMs for a survival outcome, but these methods impose restrictions on the data‐generating mechanism. Here, we propose a method that overcomes these restrictions. The MSM can be, for example, a marginal structural logistic model for a discrete survival time or a Cox or additive hazards MSM for a continuous survival time. The hazard of the potential survival time can be conditional on baseline covariates, and the treatment variable can be discrete or continuous. We illustrate the use of the proposed simulation algorithm by carrying out a brief simulation study. This study compares the coverage of confidence intervals calculated in two different ways for causal effect estimates obtained by fitting an MSM via IPTW.

## 1 Introduction

In many longitudinal observational studies, a time‐varying treatment and time‐varying covariates are observed for each individual at a number of time points (“visits”) and the aim is to estimate the causal effect of treatment on survival time. Such estimation is often complicated by time‐dependent confounding (Daniel et al. 2013). Marginal structural models (MSMs) are commonly used in this setting (Clare, Dobbins, and Mattick 2019) to estimate causal effects in a way that accounts for time‐dependent confounding. An MSM is a model for the potential outcome that would arise if treatment were assigned according to a particular rule. Here, we focus on MSMs for a survival outcome, which includes Cox MSMs and their discrete‐time analogues (Hernan, Brumback, and Robins 2000; Sterne et al. 2005; Clare, Dobbins, and Mattick 2019).

Simulation studies are often used to evaluate the performance of new (and existing) statistical methods in different scenarios (Morris, White, and Crowther 2019; Friedrich and Friede 2024). It is common in such studies to generate data in such a way that the model of interest is correctly specified. It transpires this is not always simple when the model of interest is a model for potential outcomes, as is an MSM (Evans and Didelez 2024).

Methods for simulating from Cox MSMs have been proposed by Xiao, Abrahamowicz, and Moodie (2010) and Young et al. (2010). Havercroft and Didelez (2012) and Young and Tchetgen (2014) propose algorithms for simulating from, respectively, logistic MSMs and discrete‐time Cox MSMs. By finely discretizing time, these two algorithms can be used to simulate a continuous failure time from a Cox MSM. Keogh et al. (2021) describe how to simulate from an additive‐hazards MSM.

These existing methods impose restrictions on the data‐generating mechanism. Xiao et al. (2010) rely on the failure rate being very low and do not allow for (possibly unobserved) common causes of the time‐dependent confounders and survival process, and (as noted by Young and Tchetgen 2014) the hazard in the implied MSM depends on all past treatments. Havercroft and Didelez (2012) assume the dependence of the survival process on the time‐dependent confounder process arises entirely through the effect of the latter on the treatment process and through a single shared latent variable. This means, for example, that the effect of confounders measured at time 1 on the hazard of failure at time 5, say, is as strong as their effect on the hazard at time 2, rather than being able to diminish over time. Young et al. (2010) seek to generate data in such a way that a Cox MSM, a structural nested accelerated failure time model, and a structural nested failure time model are all correctly specified, so that performance of these three modeling approaches can be compared. Their method is closely related to that of Havercroft and Didelez (2012) but requires that the potential treatment‐free survival time be exponentially distributed and the hazard in the Cox MSM depend only on the most recent treatment; also treatment must be binary. Young and Tchetgen Tchetgen (2014) aim to simulate data in such a way that three other models are correctly specified: a Cox MSM, a parametric model for the treatment given the past, and a parametric model for the hazard given the past. This allows the comparison of two methods for fitting Cox MSMs: inverse probability of treatment weighting (IPTW) and g‐computation. Their data‐generating mechanism assumes there are no baseline confounders and only one time‐dependent confounder, that the hazard at time t depends only on the treatment at the two most recent times and the time‐dependent confounder at the most recent time, and that the time‐dependent confounder at time t depends on the history of treatment and confounder only through treatment at the most recent time. In addition, none of these four data‐simulation methods explicitly allows for an MSM that conditions the hazard on baseline covariates. Keogh et al.'s (2021) proposal for simulating from an additive‐hazards MSM does not impose such restrictions but also does not enable the user easily to specify the values of the parameters of the MSM directly.

In this article, we propose an algorithm for simulating from an MSM for a survival time outcome that overcomes these restrictions. This MSM can be, for example, a logistic MSM for a discrete survival time, or a Cox or additive hazards MSM for a continuous survival time. The hazard in the MSM can be conditional on baseline covariates, and the treatment variable can be discrete or continuous. In developing our algorithm, we have broadly followed the general approach proposed by Evans and Didelez (2024) for simulating from causal models. They sketched how this approach might be used to generate from an MSM for survival data but provided few details and then only for the case of one binary confounder with a strong Markov property (see our Supporting Information Section A for details).

In Section 2, we introduce notation, define the MSM of interest, and present the causal directed acyclic graph (DAG) assumed to apply. Section 3 describes our proposed algorithm when the survival time is discrete. It requires the specification of a “risk score” function and the correlation parameter of a Gaussian copula. The risk score is a function of the history of the confounders, and the copula expresses the association between this risk score and the potential survival outcome. Application of the algorithm requires the cumulative distribution function (CDF) of the risk score to be known, which it will not be in general. So, in Section 4, we extend our algorithm to estimate this CDF at the same time as simulating the data. Section 5 describes how the algorithm can be adapted for a continuous failure time; this enables simulation from a Cox or additive hazards MSM. A brief simulation study is presented in Section 6, to illustrate the methods described in this article. R code for implementing this study is provided in the Supporting Information (or at https://github.com/shaun2022/simulatingMSM), along with Supporting Information Sections A–M. Section 7 contains a discussion.

## 2 Setup and Notation

Consider a study in which n individuals are observed at regular visits up to the earlier of their failure time and censoring time. Visit times, assumed to be the same for all individuals, are 0,1,…K, and the administrative censoring time is K+1 (which we shall sometimes call “visit K+1”). We denote random variables with capital letters and their values with lower‐case letters.

**FIGURE 1:**
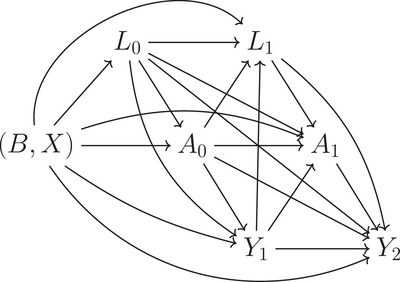
Assumed causal directed acyclic graph (DAG). For simplicity, this is shown for K=1.

Let $A_{k}$ denote the treatment received by an individual at visit k (k=0,…,K). This could be discrete or continuous. Let T denote the individual's failure time and $Y_{k}=I\left(T\geq k\right)$ be an indicator of survival to visit k (k=1,…,K+1), with $Y_{0}=1$. Let X and B denote two distinct vectors of baseline covariates for an individual. Variables X are baseline confounders and/or treatment effect modifiers that will be conditioned on in the MSM presented below (Equation 1). Variables B are not conditioned on in the MSM and can include any or all of baseline confounders, common causes of the $L_{k}$ and $Y_{k}$ variables that are not confounders, and instrumental variables. Either of X and B could be empty. Let $L_{k}$ denote time‐varying confounders for an individual at visit k (k=0,…,K). Let ${\bar{A}}_{k}=\left(A_{0},\text{…},A_{k}\right)$ and ${\bar{L}}_{k}=\left(L_{0},\text{…},L_{k}\right)$ denote the histories of treatment and time‐dependent confounders up to visit k; and ${\bar{A}}_{-1}={\bar{L}}_{-1}=\emptyset$. We assume the causal DAG shown in Figure 1.

Variables with a superscript ${\bar{a}}_{k}$ are potential variables under an intervention that sets treatments ${\bar{A}}_{k}$ at the first k+1 visits equal to ${\bar{a}}_{k}$. For example, $Y_{k+1}^{{\bar{a}}_{k}}=1$ if the individual would survive to visit k+1 if their treatments at visits 0,…,k were set to ${\bar{a}}_{k}$, and $Y_{k+1}^{{\bar{a}}_{k}}=0$ if the individual would fail. We make the usual consistency assumption that $Y_{k}=Y_{k}^{{\bar{A}}_{k-1}}$ and $L_{k}=L_{k}^{{\bar{A}}_{k-1}}$. Note that this together with the causal DAG implies sequential exchangeability, that is, $\left(Y_{k+1}^{{\bar{A}}_{k-1}\;{\underline{a}}_{k}},\text{…},Y_{K+1}^{{\bar{A}}_{k-1}\;{\underline{a}}_{k}}\right)⊥\;\;\;⊥A_{k}|X,B,{\bar{L}}_{k},{\bar{A}}_{k-1},Y_{k}=1$ for all ${\underline{a}}_{k}=\left(a_{k},\text{…},a_{K}\right)$.

We begin with MSMs for discrete‐time hazard of failure, postponing consideration of MSMs for continuous‐time hazard, for example, Cox MSMs, until Section 5. Consider the following MSM for the hazard at visit k (k=1,…,K+1).

(1) $$P\left(Y_{k}^{{\bar{a}}_{k-1}}=0|X,Y_{k-1}^{{\bar{a}}_{k-2}}=1\right)=g_{k}\left({\bar{a}}_{k-1},X;\beta \right),$$

where $g_{k}\left(.\right)$ is a known function with parameters β. Parameters β can be specific to visit k, common to all k, or a mixture of both. For example, in a logistic MSM (Robins, Hernan, and Brumback 2000), $g_{k}\left({\bar{a}}_{k-1},X;\beta \right)$ could be chosen to be, for example, $\text{expit}(\beta_{k0}+\beta_{1}^{⊤}X+\beta_{2}a_{k-1})$ or $\text{expit}(\beta_{k0}+\beta_{k1}^{⊤}X+\beta_{k2}a_{k-2}+\beta_{k3}a_{k-1}+\beta_{k4}Xa_{k-1})$.

We shall describe a method for simulating data on $\left(X,B,L_{0},A_{0},Y_{1},\text{…},L_{K},A_{K},Y_{K+1}\right)$ for a single individual such that Equation (1) is satisfied for a prespecified choice of functions $g_{k}\left({\bar{a}}_{k-1},X;\beta \right)$ (k=1,…,K+1) and value of β.

## 3 Algorithm for Simulating Data

For simplicity, suppose K=1; we shall consider K≥1 in Section 3.3. The joint distribution of $\left(X,B,L_{0},A_{0},Y_{1},L_{1},A_{1},Y_{2}\right)$ can be factorized as

(2) $$\begin{array}{ccc} & & p(X,B,L_{0},A_{0},Y_{1},L_{1},A_{1},Y_{2})\\ & & \;=p\left(X\right)\;P\left(B,L_{0}|X\right)\;P\left(A_{0}|X,B,L_{0}\right)\\ & & \;\times p\left(Y_{1}|X,B,L_{0},A_{0}\right)\;p\left(L_{1}|X,B,L_{0},A_{0},Y_{1}\right)\\ & & \;\times p(A_{1}|X,B,L_{0},A_{0},Y_{1},L_{1})\\ & & \;\times p(Y_{2}|X,B,L_{0},A_{0},Y_{1},L_{1},A_{1}). \end{array}$$

If we specify the distributions on the right‐hand side of Equation (2), we can sample $\left(X,B,L_{0},A_{0},Y_{1},L_{1},A_{1},Y_{2}\right)$. A complication is that, by the consistency assumption, $Y_{1}=Y_{1}^{A_{0}}$ and $Y_{2}=Y_{2}^{{\bar{A}}_{1}}$. This means that, for the MSM of Equation (1) to be correctly specified for the chosen functions $g_{k}\left({\bar{a}}_{k-1},X;\beta \right)$ (k=1,2) and value of β, we must choose $p(Y_{1}|X,B,L_{0},A_{0})$ and $p(Y_{2}|X,B,L_{0},A_{0},Y_{1},L_{1},A_{1})$—or equivalently, $p(Y_{1}^{A_{0}}|X,B,L_{0},A_{0})$ and $p(Y_{2}^{{\bar{A}}_{1}}|X,B,L_{0},A_{0},Y_{1},L_{1},A_{1})$—carefully. For example, sampling $Y_{1}$ from a logistic regression model with covariates X, B, $L_{0}$, and $A_{0}$ will typically not satisfy Equation (1). We now describe a suitable method for sampling $Y_{1}$ and $Y_{2}$.

### 3.1 Sampling $Y_{1}$ Given $\left(X,B,L_{0},A_{0}\right)$

Consider the task of sampling $Y_{1}^{a_{0}}$ given $\left(X,B,L_{0},A_{0}\right)$ for any value of $a_{0}$. If we can do this, then we can sample $Y_{1}^{A_{0}}$ specifically and set Y equal to it. Let $p(Y_{1}^{a_{0}}|X,B,L_{0},A_{0})$ denote a conditional distribution that satisfies the sequential exchangeability assumption, that is $p\left(Y_{1}^{a_{0}}|X,B,L_{0},A_{0}\right)=p\left(Y_{1}^{a_{0}}|X,B,L_{0}\right)$. If it also satisfies

(3) $$\begin{array}{ccc} \int P(Y_{1}^{a_{0}} & = & 0|X,B,L_{0})\;p\left(B,L_{0}|X\right)\;dB\;dL_{0}\\ & = & P\left(Y_{1}^{a_{0}}=0|X\right)=g_{1}\left(a_{0},X;\beta \right), \end{array}$$

then Equation (1) will hold for k=1. Our way of choosing a $P(Y_{1}^{a_{0}}=0|X,B,L_{0})$ that satisfies Equation (3) involves two components. The first will allow us to reduce the dependence of $Y_{1}^{a_{0}}$ on B and $L_{0}$ to dependence on a scalar function of those variables, which we call a “risk score.” The second is a bivariate Gaussian copula (Schepsmeier and Stober 2014) that will describe the association between this risk score and a latent continuous variable (denoted $U_{Y_{1}^{a_{0}}}$ below) that determines $Y_{1}^{a_{0}}$. Readers unfamiliar with Gaussian copulas can see Supporting Information Section B for an introduction.

Let $h_{0}^{a_{0}}\left(x,b,l_{0}\right)$ be a scalar continuous function of $\left(x,b,l_{0}\right)$ that the user of our algorithm should specify. We call this the “risk score” function for visit 0 because it will rank individuals with the same value of X but different values of $\left(B,L_{0}\right)$ according to their potential hazards $P(Y_{1}^{a_{0}}=0|X,B,L_{0})$, and we call $H_{0}^{a_{0}}=h_{0}^{a_{0}}\left(X,B,L_{0}\right)$ the “risk score” (for visit 0) for an individual (under the intervention that sets $A_{0}=a_{0}$). Let $F_{H_{0}^{a_{0}}}\left(h|x\right)$ denote the conditional CDF of $H_{0}^{a_{0}}$ given X=x, and let $U_{H_{0}^{a_{0}}}=F_{H_{0}^{a_{0}}}\left(H_{0}^{a_{0}}|X\right)$ be the random variable obtained by plugging $H_{0}^{a_{0}}$ and X into this CDF. We call $U_{H_{0}^{a_{0}}}$ the individual's “risk quantile.” Notice that by a general property of CDFs, $U_{H_{0}^{a_{0}}}|X∼\text{Uniform}\left(0,1\right)$.

Let Φ(.) denote the CDF of the standard normal distribution and $\rho_{0}$ ($-1<\rho_{0}\leq 0$) be some constant that the user of our algorithm will choose (in principle, $\rho_{0}$ could be a function of $a_{0}$, but, for simplicity, we assume it is not). We shall show that

(4) $$P\left(Y_{1}^{a_{0}}=0|X,B,L_{0}\right)=\Phi \left(\frac{\Phi^{-1}\left\{g_{1}\left(a_{0},X;\beta \right)\right\}-\rho_{0}\Phi^{-1}\left(U_{H_{0}^{a_{0}}}\right)}{\sqrt{1-\rho_{0}^{2}}}\right)$$

satisfies Equation (3) and that the algorithm presented below samples $Y_{1}^{a_{0}}$ from this distribution.

**FIGURE 2:**
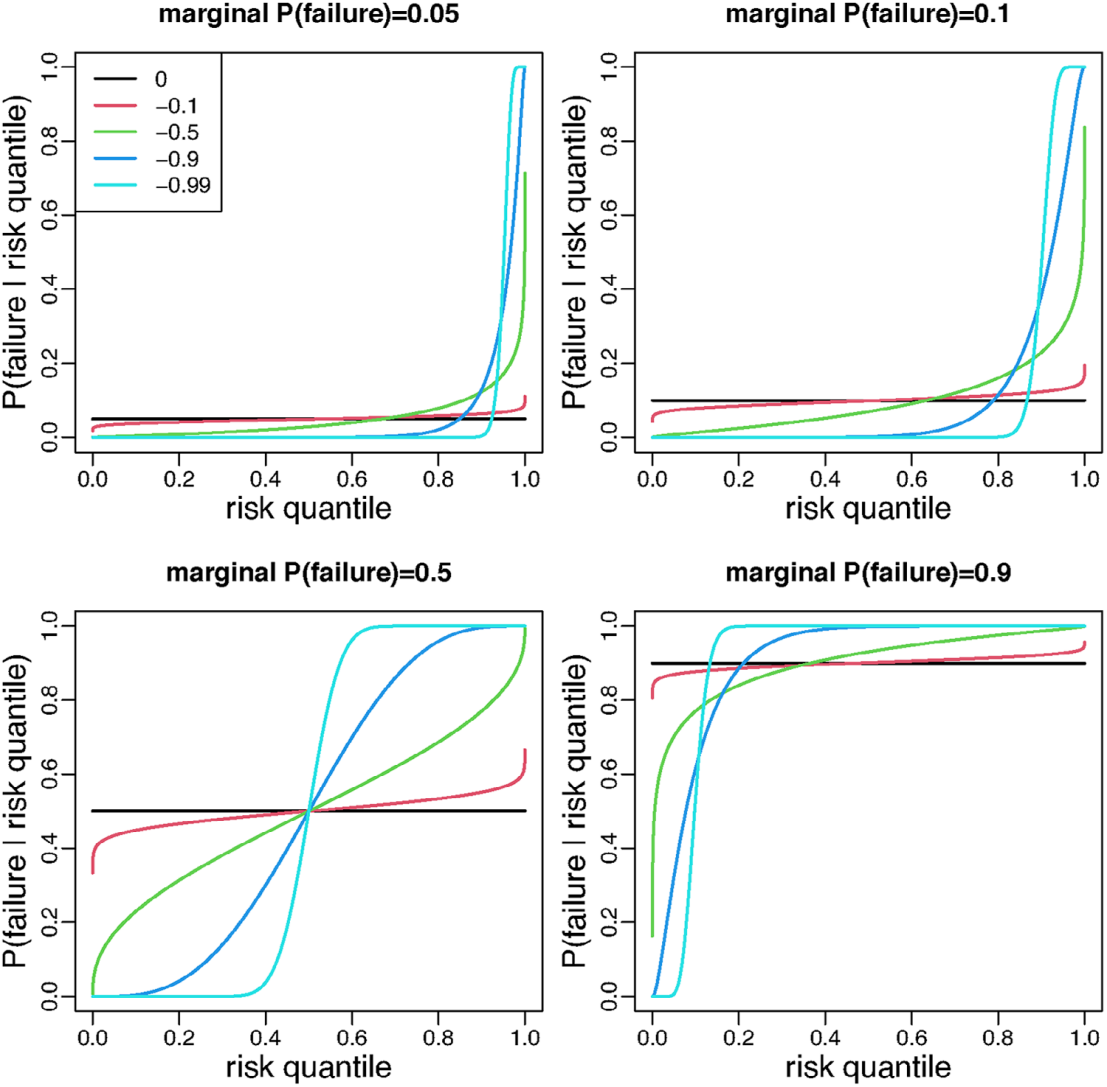
Probability $P(Y_{1}^{a_{0}}=0|X,B,L_{0})$ of failure as a function of the risk quantile $U_{H_{0}^{a_{0}}}$ for five different values of $\rho_{0}$ and four different marginal probabilities of failure $g_{1}\left(a_{0},X;\beta \right)$.

Note that $U_{H_{0}^{a_{0}}}$, and hence $P(Y_{1}^{a_{0}}=0|X,B,L_{0})$, does not change if the risk score function $h_{0}^{a_{0}}\left(x,b,l_{0}\right)$ is replaced by $\nu \{h_{0}^{a_{0}}\left(x,b,l_{0}\right)\}$, where ν(.) is any monotonically increasing function. What matters therefore is only the *ranking* of individuals that $h_{0}^{a_{0}}\left(x,b,l_{0}\right)$ provides. This and the fact that the right‐hand side of Equation (4) is an increasing function of $U_{H_{0}^{a_{0}}}$, which itself is uniformly distributed (given X), justifies naming $U_{H_{0}^{a_{0}}}$ the “risk quantile.” Parameter $\rho_{0}$ describes the strength of dependence of $P(Y_{1}^{a_{0}}=0|X,B,L_{0})$ on $U_{H_{0}^{a_{0}}}$. At one extreme, there is no dependence when $\rho_{0}=0$; at the other, $Y_{1}^{a_{0}}\approx I\left\{U_{H_{0}^{a_{0}}}<g_{1}\left(a_{0},X;\beta \right)\right\}$ when ρ≈−1 (see Figure 2).

An example of a possible risk score function is $h_{0}^{a_{0}}\left(x,b,l_{0}\right)=1^{⊤}l_{0}$, that is, the sum of the elements of the vector $l_{0}$. This risk score function means that of two individuals who have the same value of X but different values of $\left(B,L_{0}\right)$, the individual with the higher value of $1^{⊤}L_{0}$ has the greater potential hazard (when $A_{0}$ is set to $a_{0}$ by an intervention). The reason why $h_{0}^{a_{0}}\left(x,b,l_{0}\right)$ is written with a superscript $a_{0}$ and as a function of x is that the user can, if desired, specify that the dependence of $P(Y_{1}^{a_{0}}=0|X,B,L_{0})$ on B and $L_{0}$ is different for different values of $a_{0}$ and/or X. For example, if $A_{0}$ is binary and $h_{0}^{0}\left(x,b,l_{0}\right)=l_{0}$ and $h_{0}^{1}\left(x,b,l_{0}\right)=0$, then $L_{0}$ affects an individual's potential hazard only if treatment is set to zero.

Our algorithm involves the following steps. First, calculate $H_{0}^{a_{0}}$, then $U_{H_{0}^{a_{0}}}=F_{H_{0}^{a_{0}}}\left(H_{0}^{a_{0}}|X\right)$ and $Z_{H_{0}^{a_{0}}}=\Phi^{-1}\left(U_{H_{0}^{a_{0}}}\right)$. Then generate $Z_{Y_{1}^{a_{0}}}∼\text{Normal}\left(\rho_{0}Z_{H_{0}^{a_{0}}},1-\rho_{0}^{2}\right)$ and calculate $U_{Y_{1}^{a_{0}}}=\Phi \left(Z_{Y_{1}^{a_{0}}}\right)$. Finally, set

(5) $$Y_{1}^{a_{0}}=0\;\text{if}\;U_{Y_{1}^{a_{0}}}<g_{1}\left(a_{0},X;\beta \right)\;\text{and}\;Y_{1}^{a_{0}}=1\;\text{otherwise}.$$

We now show that this algorithm generates $Y_{1}^{a_{0}}$ from Equation (4) and satisfies Equation (1) for k=1. First, notice that $Z_{H_{0}^{a_{0}}}|X∼\text{Normal}\left(0,1\right)$, and hence that $\left(Z_{H_{0}^{a_{0}}},Z_{Y_{1}^{a_{0}}}\right)$ has a bivariate normal distribution given X with means 0, variances 1, and correlation $\rho_{0}$. This implies $U_{Y_{1}^{a_{0}}}|X∼\text{Uniform}\left(0,1\right)$. It follows from this and expression (5) that Equation (1) is satisfied for k=1. Also, Equation (4) immediately follows from

$$\begin{array}{ccc} & & P\left(Y_{1}^{a_{0}}=0|X,B,L_{0}\right)=P\left(U_{Y_{1}^{a_{0}}}<g_{1}\left(a_{0},X;\beta \right)|X,B,L_{0}\right)\\ & & \;=P\left(\frac{\Phi^{-1}\left(U_{Y_{1}^{a_{0}}}\right)-\rho_{0}Z_{H_{0}^{a_{0}}}}{\sqrt{1-\rho_{0}^{2}}}<\frac{\Phi^{-1}\left\{g_{1}\left(a_{0},X;\beta \right)\right\}-\rho_{0}Z_{H_{0}^{a_{0}}}}{\sqrt{1-\rho_{0}^{2}}}|X,B,L_{0}\right). \end{array}$$

Readers familiar with copulas will recognize that we have used a Gaussian copula to describe the conditional association between $U_{H^{a_{0}}}$ and $U_{Y_{1}^{a_{0}}}$ given X, and sampled $U_{Y_{1}^{a_{0}}}$ from its conditional distribution given $\left(U_{H^{a_{0}}},X\right)$ implied by this copula.

### 3.2 Sampling $Y_{2}$ Given $\left(X,B,L_{0},A_{0},Y_{1},L_{1},A_{1}\right)$

Suppose we have generated $Y_{1}=Y_{1}^{A_{0}}$. If $Y_{1}=0$, stop: the individual has failed before visit 1. Otherwise, sample $Y_{2}$ given $\left(X,B,L_{0},A_{0},L_{1},A_{1}\right)$ and $Y_{1}=1$ using the procedure we now describe, a procedure analogous to that used to generate $Y_{1}$. This time, we need to specify (i) a risk score function that ranks individuals with the same value of X and $Y_{1}^{a_{0}}=1$ but different values of $\left(B,L_{0},L_{1}^{a_{0}}\right)$ by their hazards and (ii) the correlation parameter $\rho_{1}$ of a Gaussian copula for the conditional association (given X and $Y_{1}^{a_{0}}=1$) between the risk quantile and a latent continuous variable $U_{Y_{2}^{{\bar{a}}_{1}}}$ that determines $Y_{2}^{{\bar{a}}_{1}}$.

Consider the task of generating $Y_{2}^{A_{0}a_{1}}$ given $\left(X,B,L_{0},A_{0},L_{1},A_{1}\right)$ and $Y_{1}=1$ for any value of $a_{1}$. If we can do this, then we can generate $Y_{2}^{{\bar{A}}_{1}}$ specifically and set $Y_{2}$ equal to it. The causal DAG and consistency assumption together imply $P\left(Y_{2}^{{\bar{a}}_{1}}=0|X,B,L_{0},{\bar{A}}_{1}={\bar{a}}_{1},Y_{1}=1,L_{1}\right)=P\left(Y_{2}^{{\bar{a}}_{1}}=0|X,B,L_{0},Y_{1}^{a_{0}}=1,L_{1}^{a_{0}}\right)$ for any ${\bar{a}}_{1}$ (see Supporting Information Section C for proof). When, as here, $a_{0}$ is the previously generated value of $A_{0}$, the consistency assumption implies that $L_{1}^{a_{0}}=L_{1}$ and $Y_{1}^{a_{0}}=Y_{1}$ are known (i.e., have been previously generated).

Let $h_{1}^{{\bar{a}}_{1}}=h_{1}^{{\bar{a}}_{1}}\left(x,b,l_{0},l_{1}\right)$ be a scalar continuous function of $\left(x,b,l_{0},l_{1}\right)$, specified by the user. This is the “risk score” function for visit 1, and $H_{1}^{{\bar{a}}_{1}}=h_{1}^{{\bar{a}}_{1}}\left(X,B,L_{0},L_{1}^{a_{0}}\right)$ is the individual's “risk score” (for visit 1). Examples of possible $h_{1}^{{\bar{a}}_{1}}\left(x,b,l_{0},l_{1}\right)$ are $1^{⊤}l_{1}$ and $1^{⊤}\left(l_{1}-l_{0}\right)$. The latter implies that the potential hazard of failure (when ${\bar{A}}_{1}$ is set to ${\bar{a}}_{1}$ by an intervention) depends on the most recent change in the values of the time‐dependent confounders. Let $F_{H_{1}^{{\bar{a}}_{1}}}\left(h|x,Y_{1}^{a_{0}}=1\right)$ denote the CDF of $H_{1}^{{\bar{a}}_{1}}$ given X=x and $Y_{1}^{a_{0}}=1$, and call $U_{H_{1}^{{\bar{a}}_{1}}}=F_{H_{1}^{{\bar{a}}_{1}}}\left(H_{1}^{{\bar{a}}_{1}}|X,Y_{1}^{a_{0}}=1\right)$ the individual's “risk quantile” (for visit 1).

Let $Z_{H_{1}^{{\bar{a}}_{1}}}=\Phi^{-1}\left(U_{H_{1}^{{\bar{a}}_{1}}}\right)$ and generate $Z_{Y_{2}^{{\bar{a}}_{1}}}∼\text{Normal}\left(\rho_{1}Z_{H_{1}^{{\bar{a}}_{1}}},1-\rho_{1}^{2}\right)$. Calculate $U_{Y_{2}^{{\bar{a}}_{1}}}=\Phi \left(Z_{Y_{2}^{{\bar{a}}_{1}}}\right)$ and set $Y_{2}^{{\bar{a}}_{1}}=0$ if $U_{Y_{2}^{{\bar{a}}_{1}}}<g_{2}\left({\bar{a}}_{1},X;\beta \right)$ and $Y_{2}^{{\bar{a}}_{1}}=1$ otherwise. This ensures that Equation (1) is satisfied for k=2, and $Y_{2}^{{\bar{a}}_{1}}$ is generated from

$$\begin{array}{ccc} & & P(Y_{2}^{{\bar{a}}_{1}}=0|X,B,L_{0},Y_{1}^{a_{0}}=1,L_{1}^{a_{0}})\\ & & \;=\Phi \left(\frac{\Phi^{-1}\left\{g_{2}\left({\bar{a}}_{1},X;\beta \right)\right\}-\rho_{1}\Phi^{-1}\left(U_{H_{1}^{{\bar{a}}_{1}}}\right)}{\sqrt{1-\rho_{1}^{2}}}\right). \end{array}$$

### 3.3 Generalization to More Than Two Visits

This method generalizes to K≥1. First, specify distributions p(X) and p(B∣X), and $p(L_{k}|X,B,{\bar{L}}_{k-1},{\bar{A}}_{k-1},Y_{k}=1)$ and $p(A_{k}|X,B,{\bar{L}}_{k},{\bar{A}}_{k-1},Y_{k}=1)$ for each k=0,…,K. Also for each k=0,…,K, specify a scalar continuous risk score function $h_{k}^{{\bar{a}}_{k}}\left(x,b,{\bar{l}}_{k}\right)$ and correlation parameter $-1<\rho_{k}\leq 0$ of a Gaussian copula for the conditional association between risk quantile $U_{H_{k}^{{\bar{a}}_{k}}}$ and latent variable $U_{Y_{k+1}^{{\bar{a}}_{k}}}$ (both defined below) given X and $Y_{k}^{{\bar{a}}_{k-1}}=1$. The risk score function will rank individuals with the same value of X and $Y_{k}^{{\bar{a}}_{k-1}}=1$ but different values of $\left(B,{\bar{L}}_{k}^{{\bar{a}}_{k-1}}\right)$. Now,

1. Sample X from p(X) and then B from p(B∣X). Set k=0.
2. Sample $L_{k}$ from $p(L_{k}|X,B,{\bar{L}}_{k-1},{\bar{A}}_{k-1},Y_{k}=1)$.
3. Sample $A_{k}$ from $p(A_{k}|X,B,{\bar{L}}_{k},{\bar{A}}_{k-1},Y_{k}=1)$ and call the result $a_{k}$.
4. Calculate $H_{k}^{{\bar{a}}_{k}}=h_{k}^{{\bar{a}}_{k}}\left(X,B,{\bar{L}}_{k}\right)$.
5. Calculate $U_{H_{k}^{{\bar{a}}_{k}}}=F_{H_{k}^{{\bar{a}}_{k}}}\left(H_{k}^{{\bar{a}}_{k}}|X,Y_{k}^{{\bar{a}}_{k-1}}=1\right)$ and $Z_{H_{k}^{{\bar{a}}_{k}}}=\Phi^{-1}\left(U_{H_{k}^{{\bar{a}}_{k}}}\right)$.
6. Sample $Z_{Y_{k+1}^{{\bar{a}}_{k}}}∼\text{Normal}\left(\rho_{k}Z_{H_{k}^{{\bar{a}}_{k}}},1-\rho_{k}^{2}\right)$ and calculate $U_{Y_{k+1}^{{\bar{a}}_{k}}}=\Phi \left(Z_{Y_{k+1}^{{\bar{a}}_{k}}}\right)$.
7. If $U_{Y_{k+1}^{{\bar{a}}_{k}}}<g_{k+1}\left({\bar{a}}_{k},X;\beta \right)$, set $Y_{k+1}=0$. Otherwise set $Y_{k+1}=1$.
8. If $Y_{k+1}=1$ and k<K, let k=k+1 and return to step 2.

Note that we set $H_{k}^{{\bar{a}}_{k}}=h_{k}^{{\bar{a}}_{k}}\left(X,B,{\bar{L}}_{k}\right)$ at step 4 because the consistency assumption means that ${\bar{L}}_{k}^{{\bar{a}}_{k}}={\bar{L}}_{k}$ for this value of ${\bar{a}}_{k}$, and we are implicitly setting $Y_{k+1}=Y_{k+1}^{{\bar{a}}_{k}}$ at step 7 for the same reason. Note also that this algorithm does not require the rejection sampling that Evans and Didelez (2024) use in general (Seaman 2024).

## 4 Extended Algorithm for Estimating CDF of Risk Score and Simulating Data

For a limited range of data‐generating mechanisms, the CDF $F_{H_{k}^{{\bar{a}}_{k}}}\left(h|x,Y_{k}^{{\bar{a}}_{k-1}}=1\right)$ is known (see the final paragraph of Supporting Information Section A). In general, however, it is not, which poses an obstacle to implementing the algorithm of Section 3. When the number of possible values of $\left(X,{\bar{A}}_{K}\right)$ is small, $F_{H_{k}^{{\bar{a}}_{k}}}\left(h|x,Y_{k}^{{\bar{a}}_{k-1}}=1\right)$ can be estimated separately for each of these values using the procedure described in Supporting Information Section D. More generally, one can use the following extended algorithm, which involves simultaneously generating data and estimating the CDFs. This is done by generating data not only for a single sampled individual but also for a moderately large number (here we use 4999) of matching individuals at the same time. Each of these matches has the same values of X and ${\bar{A}}_{K}$ as the sampled individual, but its values of B, $L_{k}$, and $Y_{k}$ are randomly generated (in the same way as for the sampled individual), and so will usually differ from the sampled individual's values. These 5000 individuals, that is, the one sampled individual and that individual's 4999 matches, are used to estimate their values of $F_{H_{k}^{{\bar{a}}_{k}}}\left(H_{k}^{{\bar{a}}_{k}}|X,Y_{k}^{{\bar{a}}_{k-1}}=1\right)$ immediately before sampling their $Y_{k+1}$ values. Once the data for the sampled individual have been generated, the data on the 4999 matches are discarded.

The algorithm for generating data for a *single* sampled individual is as follows. In this algorithm, the single sampled individual and the 4999 matches of this individual are indexed by j, with j=1 being the sampled individual and j=2,…,m being the matches, where m=5000. A random variable not mentioned so far, $I_{j}$ (j=2,…,m), appears in the algorithm. We shall make other comments about the algorithm after stating it.

1. Sample $X_{1}$ from p(X). Let $X_{j}=X_{1}$ and $I_{j}=j$ for j=2,…,m. Set k=0.
2. For j=1,…,m, sample $B_{j}$ from $p(B_{j}|X_{j})$.
3. For j=1,…,m, sample $L_{kj}$ from $p(L_{kj}|X_{j},B_{j},{\bar{L}}_{k-1,j},{\bar{A}}_{k-1,1}={\bar{a}}_{k-1},Y_{kj}=1)$.
4. Sample $A_{k1}$ from $p(A_{k1}|X_{1},B_{1},{\bar{L}}_{k1},{\bar{A}}_{k-1,1}={\bar{a}}_{k-1},Y_{k1}=1)$ and call the result $a_{k}$.
5. For j=1,…,m, calculate $H_{kj}=h_{k}^{{\bar{a}}_{k}}\left(X_{j},B_{j},{\bar{L}}_{kj}\right)$.
6. For j=1,…,m, Let $R_{kj}$ denote the rank of $H_{kj}$ among the set $\left\{H_{k1},\text{…},H_{km}\right\}$, sample $W_{kj}∼\text{Uniform}\left(0,1\right)$, and calculate $U_{H_{k}^{{\bar{a}}_{k}},j}=\left(R_{kj}-W_{kj}\right)/m$.
7. For j=1,…,m, calculate $Z_{H_{k}^{{\bar{a}}_{k}},j}=\Phi^{-1}\left(U_{H_{k}^{{\bar{a}}_{k}},j}\right)$.
8. For j=1,…,m, Sample $Z_{Y_{k+1}^{{\bar{a}}_{k}},j}∼\text{Normal}\left(\rho_{k}Z_{H_{k}^{{\bar{a}}_{k}},j},1-\rho_{k}^{2}\right)$ and calculate $U_{Y_{k+1}^{{\bar{a}}_{k}},j}=\Phi \left(Z_{Y_{k+1}^{{\bar{a}}_{k}},j}\right)$.
9. For j=1,…,m, set $Y_{k+1,j}=0$ if $U_{Y_{k+1}^{{\bar{a}}_{k}},j}<g_{k+1}\left({\bar{a}}_{k},X_{j};\beta \right)$ and set $Y_{k+1,j}=1$ otherwise.
10. If $Y_{k+1,1}=0$ or k=K, stop.
11. For j=2,…,m, if $Y_{k+1,j}=0$, randomly choose $2\leq j^{*}\leq m$ such that $Y_{k+1,j^{*}}=1$, and set $I_{j}=I_{j^{*}}$, $B_{j}=B_{j^{*}}$, ${\bar{L}}_{kj}={\bar{L}}_{kj^{*}}$ and $Y_{k+1,j}=1$.
12. Let k=k+1 and return to step 3.

Readers wishing to understand this algorithm should note that $\left\{H_{k1},\text{…},H_{km}\right\}$ in step 6 is a random sample from the distribution of $H_{k}^{{\bar{a}}_{k-1}}$ given X and $Y_{k}=1$, and hence $R_{kj}/m$ is an estimate of $F_{H_{k}^{{\bar{a}}_{k}}}\left(H_{kj}^{{\bar{a}}_{k}}|X_{j},Y_{kj}^{{\bar{a}}_{k-1}}=1\right)$. The subtraction of $W_{kj}$ from $R_{kj}$ in step 6 ensures $U_{H_{k}j}^{{\bar{a}}_{k}}$ given $X_{j}$ and $Y_{kj}=1$ is Uniform(0,1).

The purpose of step 11 is to replace each match that fails at time k+1 with a copy of a randomly chosen match that has not yet failed, thus maintaining the number of matches at m−1=4999. Variables $I_{2},\text{…},I_{m}$ keep track of which matches are copies of which other matches. At first, $I_{j}=j$ for all j (see step 1), but each time a match, say match j, fails and is replaced by a copy of another match, say match $j^{*}$, $I_{j}$ is changed to $j^{*}$ (step 11).

A potential problem is that eventually the number of distinct values of $\left\{I_{2},\text{…},I_{m}\right\}$ could be small, indicating that few of the original 4999 matches remain. This could cause the algorithm to perform poorly, because it would mean, for example, that the matches only had a small number of distinct values of $\left(B,L_{1}\right)$. This is most likely to happen when $P(Y_{k}^{{\bar{a}}_{k-1}}=0|X)$ for the sampled individual's value of X is close to one. To address this issue, we add an extra step that discards the existing matches and restarts with a much larger number (we use m=100,000) of matches whenever few (e.g., less than 10%) of the original matches remain at step $k^{*}+1$. The data generated for the sampled individual (j=1) up to visit $k^{*}$ and their indicator, $Y_{k^{*}+1,1}$, of survival to visit $k^{*}+1$ are kept. (See Supporting Information Section E for details.)

In Supporting Information Section F, we demonstrate that this algorithm does indeed generate data compatible with the chosen MSM, by simulating data on one million individuals, fitting the MSM to these data, and verifying that the parameter estimates obtained are very close to the true parameter values. We also present (Supporting Information Section G) an investigation of sensitivity of results to the choice of m, where we find that m=1000 in steps 1–12 and m=20,000 in the extra step is probably adequate.

## 5 Continuous Failure Time

Extending our algorithms to simulate data for a continuous‐time MSM, for example, Cox or additive hazards MSM, is straightforward. This MSM specifies $\lambda^{{\bar{a}}_{k}}\left(t|X=x;\beta \right)$, the continuous‐time conditional hazard at time t (k<t≤k+1) of the potential failure time $T^{{\bar{a}}_{k}}$ given X=x when we intervene to set ${\bar{A}}_{k}={\bar{a}}_{k}$. For example, in a Cox MSM, $\lambda^{{\bar{a}}_{k}}\left(t|X=x;\beta \right)$ (for k<t≤t+1) could be chosen to be, for example, $\lambda^{{\bar{0}}_{K}}\left(t|X=0\right)\exp\left(\beta_{1}^{⊤}x+\beta_{2}a_{k}\right)$ or $\lambda^{{\bar{0}}_{K}}\left(t|X=0\right)\exp\left(\beta_{k1}^{⊤}x+\beta_{k2}a_{k-1}+\beta_{k3}a_{k}+\beta_{k4}xa_{k}\right)$, where $\lambda^{{\bar{0}}_{K}}\left(t|X=0\right)$ is the potential hazard for an individual with X=0 when all treatments are set equal to zero. An example of an additive‐hazards MSM is $\lambda^{{\bar{a}}_{k}}\left(t|X=x;\beta \right)=\lambda^{{\bar{0}}_{K}}\left(t|X=0\right)+\beta_{1}^{⊤}x+\beta_{2}a_{k}$.

When fitting an MSM, the analyst treats the β parameters as unknown quantities to be estimated. To simulate data, however, we need to fully specify $\lambda^{{\bar{a}}_{k}}\left(t|X=x;\beta \right)$, including the value of β. We also need to specify a risk score function $h_{k}^{{\bar{a}}_{k}}\left(x,b,{\bar{l}}_{k}\right)$ and a Gaussian copula correlation parameter $\rho_{k}$. In Sections 3 and 4, we assumed $P(Y_{k+1}^{{\bar{a}}_{k}}=0|X=x,B=b,{\bar{L}}_{k}^{{\bar{a}}_{k-1}}={\bar{l}}_{k},Y_{k}^{{\bar{a}}_{k-1}}=1)$ depends on x, b, and ${\bar{l}}_{k}$ only through x and $h_{k}^{{\bar{a}}_{k}}\left(x,b,{\bar{l}}_{k}\right)$, with the strength of this dependence being described by $\rho_{k}$. Now, we assume this is (also) true of $\lambda^{{\bar{a}}_{k}}\left(t|x,b,{\bar{l}}_{k}\right)$, the potential hazard at time t (k<t≤k+1) given X=x, B=b, and ${\bar{L}}_{k}^{{\bar{a}}_{k-1}}={\bar{l}}_{k}$ when we intervene to set ${\bar{A}}_{k}={\bar{a}}_{k}$. (See Supporting Information Section H for details.)

## 6 Illustrative Simulation Study

We illustrate how the proposed methods could be used in practice to carry out a simulation study. In this illustrative study, the aim is to compare the simple sandwich variance estimator and nonparametric bootstrap as two methods for calculating Confidence Intervals (CIs) and testing for treatment effect modification when fitting an MSM using IPTW. Unlike the simple sandwich estimator, bootstrap accounts for the uncertainty associated with estimating the IPT weights.

### 6.1 Methods

We simulated data for K=9, binary treatment $A_{k}$, two baseline confounders $X={\left(X_{1},X_{2}\right)}^{⊤}$ in the MSM, two baseline variables $B={\left(B_{1},B_{2}\right)}^{⊤}$ not in the MSM, two time‐dependent confounders $L_{k}={\left(L_{k1},L_{k2}\right)}^{⊤}$, and continuous failure time T. We specified $X_{1}∼\text{Normal}\left(0,1\right)$; $X_{2}|X_{1}∼\text{Bernoulli}\left(0.5\right)$; $B_{1}|X∼\text{Normal}\left(-0.2+0.4X_{2},1\right)$; $B_{2}|X,B_{1}∼\text{Normal}\left(0.2X_{1},1\right)$; $L_{01}|X,B∼\text{Normal}\left(0.2X_{1},1\right)$; $L_{02}|X,B,L_{01}∼\text{Bernoulli}\left(\text{expit}\left(-0.2+0.4X_{2}\right)\right)$; $L_{k1}|X,B,{\bar{L}}_{k-1}$, ${\bar{A}}_{k-1},Y_{k}=1∼\text{Normal}\left(0.3+0.4B_{2}+0.7L_{k-1,1}-0.6A_{k-1},1\right)$ and $L_{k2}|X,B,{\bar{L}}_{k-1},{\bar{A}}_{k-1},L_{k1},Y_{k}=1∼\text{Bernoulli}\left(\text{expit}\left(-0.2+0.4B_{2}+L_{k-1,2}-0.6A_{k-1}\right)\right)$ for k=1,…,9; and $P\left(A_{k}=1|X,B,{\bar{L}}_{k},{\bar{A}}_{k-1},Y_{k}=1\right)=\text{expit}(-1+$ $\delta_{1}X_{1}+\delta_{2}X_{2}+\delta_{3}B_{1}+\delta_{4}L_{k1}+\delta_{5}L_{k2}+A_{k-1})$ for k=0,…,9. We considered two values for $\delta =(\delta_{1},\delta_{2},\delta_{3},\delta_{4},\delta_{5})$: $\delta_{\mathrm{low}}=\left(0.1,0.15,0.1,0.3,0.3\right)$ and $\delta_{\mathrm{high}}=\left(0.2,0.3,0.2,0.6,0.6\right)$.

We generated data satisfying Cox MSM $\lambda^{{\bar{a}}_{k}}\left(t|X\right)=\lambda_{0}\left(t\right)\exp(\beta_{1}X_{1}+\beta_{2}X_{2}$ $+\beta_{3}a_{k}+\beta_{4}X_{1}a_{k}+\beta_{5}X_{2}a_{k})$ with $\beta =\left(\beta_{1},\beta_{2},\beta_{3},\beta_{4},\beta_{5}\right)=\left(0.5,0.5,-1,-0.4,0\right)$ and $\lambda_{0}\left(t\right)=\exp\left(-3.3\right)$. Since $\beta_{5}=0$, only $X_{1}$ is an effect modifier.

We chose risk score function $h_{k}^{{\bar{a}}_{k}}\left(x,b,{\bar{l}}_{k}\right)=0.3b_{1}+0.5b_{2}+l_{k1}+l_{k2}$. This means that when ${\bar{A}}_{k}$ is set to ${\bar{a}}_{k}$ by an intervention, an individual with large values of $B_{1}$, $B_{2}$, $L_{k1}$, and $L_{k2}$ has a larger hazard between times k and k+1 than does the average individual with the same value of X. We considered two values for the copula correlation $\rho_{k}$: $\rho_{\mathrm{low}}=-0.5$ (weak dependence) and $\rho_{\mathrm{high}}=-0.9$ (strong dependence). Confounding by X, $B_{1}$, and $L_{k}$ is strongest when $\rho =\rho_{\mathrm{high}}$ and $\delta =\delta_{\mathrm{high}}$, and weakest when $\rho =\rho_{\mathrm{low}}$ and $\delta =\delta_{\mathrm{low}}$. Variable $B_{2}$ is a common cause of $L_{k}$ and $Y_{k+1}^{{\bar{a}}_{k}}$ but not a confounder, because it does not (directly) influence $A_{k}$. We made $B_{2}$ an unobserved variable; our algorithm generates values for $B_{2}$, but these are not used in the data analysis.

We generated random censoring times from an exponential distribution with rate exp(−3.6). The marginal probability of observing an individual's failure time varied from 0.21 to 0.25, depending on the values of ρ and δ. The marginal probability of (random) censoring before time K+1 varied from 0.20 to 0.21.

We considered 12 scenarios, corresponding to the two values of each of ρ and δ and three sample sizes: n=250, 500, and 1000. For each scenario, we generated 1000 datasets. The Cox MSM was fitted to each dataset using either no weights (“naive method”) or IPTW. When using IPTW, (stabilized) weights at visit k were calculated in the standard way (Hernan, Brumback, and Robins 2000) using logistic regression of $A_{k}$ on $X,B_{1},L_{k}$, and ${\bar{A}}_{k-1}$ (see Supporting Information Section I for details). We calculated 95% CIs for the β parameters using the sandwich variance estimator (“sandwich CIs”) and the percentile bootstrap method (with 1000 bootstrap samples) (“bootstrap CIs”). Size‐0.05 tests of the null hypotheses of no effect modification by each of $X_{1}$ and $X_{2}$ were performed by rejecting the null if the 95% CI for, respectively, $\beta_{4}$ and $\beta_{5}$ excluded zero.

### 6.2 Results

Table 1 shows the bias of the parameter estimators. The naive estimators are biased, particularly that of $\beta_{3}$. When n=1000 and $\delta =\delta_{\mathrm{low}}$, IPTW estimators are approximately unbiased. When n=1000 and $\delta =\delta_{\mathrm{high}}$, there is some bias in the IPTW estimator of $\beta_{3}$, particularly when $\rho =\rho_{\mathrm{high}}$, but this is small compared to the true value (i.e., $\beta_{3}=-1$). Bias in the IPTW estimator of $\beta_{3}$ tends to worsen slightly when n=500 or n=250, but does not exceed 0.12.

**TABLE 1:** Bias in estimators of $\beta_{1},\text{…},\beta_{5}$ from the naive and IPTW analyses for 12 scenarios: two values of δ and ρ and three sample sizes n. The maximum Monte Carlo standard errors (SEs) for the bias in the 20 naive estimators when, respectively, n=1000, 500, and 250 are 0.009, 0.013, and 0.018. The corresponding values for the IPTW estimators are 0.015, 0.020, and 0.027.

	n=1000		n=500		n=250	
	Naive	IPTW	Naive	IPTW	Naive	IPTW
$\delta_{\mathrm{low}},\rho_{\mathrm{low}}$						
$\beta_{1}$	0.013	0.003	0.019	0.005	0.022	0.009
$\beta_{2}$	0.011	0.000	0.019	0.018	0.016	0.018
$\beta_{3}$	0.594	−0.020	0.577	−0.045	0.552	−0.069
$\beta_{4}$	−0.034	−0.005	−0.039	0.001	−0.039	−0.005
$\beta_{5}$	−0.062	0.003	−0.026	0.043	−0.032	0.020
$\delta_{\mathrm{high}},\rho_{\mathrm{low}}$						
$\beta_{1}$	0.011	0.009	0.017	0.018	0.020	0.023
$\beta_{2}$	0.003	0.004	−0.002	0.013	0.004	0.010
$\beta_{3}$	1.002	0.027	0.994	0.020	0.973	−0.009
$\beta_{4}$	−0.061	−0.010	−0.074	−0.012	−0.079	−0.025
$\beta_{5}$	−0.088	0.007	−0.083	0.001	−0.042	0.079
$\delta_{\mathrm{low}},\rho_{\mathrm{high}}$						
$\beta_{1}$	0.038	0.000	0.045	0.004	0.038	−0.001
$\beta_{2}$	0.038	0.007	0.047	0.015	0.041	0.000
$\beta_{3}$	1.001	−0.008	1.005	0.002	0.954	−0.061
$\beta_{4}$	−0.070	−0.003	−0.080	−0.014	−0.066	0.000
$\beta_{5}$	−0.105	−0.006	−0.108	−0.007	−0.093	0.035
$\delta_{\mathrm{high}},\rho_{\mathrm{high}}$						
$\beta_{1}$	0.046	0.000	0.060	0.019	0.048	0.016
$\beta_{2}$	0.026	0.025	0.039	0.036	0.052	0.051
$\beta_{3}$	1.773	0.051	1.788	0.083	1.792	0.123
$\beta_{4}$	−0.118	−0.003	−0.131	−0.013	−0.116	−0.018
$\beta_{5}$	−0.140	−0.008	−0.143	−0.001	−0.145	−0.020

Table 2 shows coverage of CIs. Coverage of naive CIs is very low for $\beta_{3}$, reflecting the bias in its naive point estimator. When n=1000 and $\delta =\delta_{\mathrm{low}}$, there is no obvious difference between coverage of sandwich and bootstrap CIs. When n=1000 and $\delta =\delta_{\mathrm{high}}$, sandwich and bootstrap CIs both tend to have slight undercoverage, but bootstrap CI coverage is closer to 95%. Results for n=500 and n=250 are qualitatively similar, with coverage deteriorating for both methods but remaining closer to 95% for bootstrap. Table 3 shows the power of the test of effect modification by $X_{1}$ (power for $X_{2}$ is one minus coverage of the CI for $\beta_{5}$). Power is sometimes greater for the sandwich method than for bootstrap, but this usually reflects undercoverage of sandwich CIs.

**TABLE 2:** Coverage of naive, sandwich, and bootstrap 95% CIs for $\beta_{1},\text{…},\beta_{5}$ for 12 scenarios: two values of δ and ρ and three sample sizes n. Naive CIs are constructed as unweighted (naive) point estimate plus/minus model‐based SE, sandwich CIs as IPTW point estimate plus/minus square root of sandwich variance estimate, and bootstrap CIs using IPTW and percentile method. When true coverage is 0.95, Monte Carlo SEs are approximately 0.007, 0.010, and 0.014 when n=1000, 500, and 250, respectively.

	n=1000			n=500			n=250		
	Naive	Sand	Boot	Naive	Sand	Boot	Naive	Sand	Boot
$\delta_{\mathrm{low}},\rho_{\mathrm{low}}$									
$\beta_{1}$	0.943	0.944	0.945	0.933	0.926	0.923	0.942	0.930	0.939
$\beta_{2}$	0.947	0.942	0.945	0.949	0.935	0.933	0.971	0.948	0.947
$\beta_{3}$	0.216	0.945	0.938	0.486	0.946	0.936	0.685	0.953	0.940
$\beta_{4}$	0.937	0.935	0.938	0.927	0.931	0.937	0.936	0.928	0.938
$\beta_{5}$	0.941	0.940	0.943	0.951	0.939	0.938	0.967	0.945	0.948
$\delta_{\mathrm{high}},\rho_{\mathrm{low}}$									
$\beta_{1}$	0.945	0.905	0.927	0.942	0.908	0.931	0.941	0.894	0.934
$\beta_{2}$	0.954	0.913	0.934	0.960	0.896	0.929	0.954	0.891	0.927
$\beta_{3}$	0.005	0.930	0.942	0.091	0.915	0.951	0.338	0.905	0.932
$\beta_{4}$	0.918	0.907	0.938	0.927	0.910	0.942	0.943	0.900	0.944
$\beta_{5}$	0.930	0.924	0.943	0.944	0.905	0.932	0.950	0.908	0.936
$\delta_{\mathrm{low}},\rho_{\mathrm{high}}$									
$\beta_{1}$	0.928	0.950	0.948	0.918	0.937	0.942	0.943	0.924	0.935
$\beta_{2}$	0.945	0.957	0.945	0.948	0.946	0.940	0.945	0.947	0.948
$\beta_{3}$	0.004	0.947	0.937	0.081	0.959	0.945	0.359	0.947	0.938
$\beta_{4}$	0.924	0.945	0.943	0.921	0.951	0.947	0.923	0.931	0.939
$\beta_{5}$	0.918	0.943	0.944	0.938	0.945	0.944	0.942	0.933	0.927
$\delta_{\mathrm{high}},\rho_{\mathrm{high}}$									
$\beta_{1}$	0.932	0.910	0.924	0.932	0.905	0.927	0.921	0.886	0.931
$\beta_{2}$	0.954	0.918	0.932	0.949	0.889	0.919	0.965	0.915	0.939
$\beta_{3}$	0.000	0.920	0.925	0.000	0.918	0.926	0.018	0.925	0.939
$\beta_{4}$	0.864	0.926	0.941	0.894	0.926	0.951	0.917	0.912	0.940
$\beta_{5}$	0.923	0.920	0.931	0.928	0.913	0.925	0.954	0.923	0.950

**TABLE 3:** Power of test of the null hypothesis that $\beta_{4}=0$ for 12 scenarios: two values of δ and ρ and three sample sizes n. The null is rejected if naive/sandwich/bootstrap 95% CI excludes zero. The maximum Monte Carlo SE for the power is 0.02, 0.02, and 0.03 when n=1000, 500, and 250, respectively.

		n=1000			n=500			n=250		
		Naive	Sand	Boot	Naive	Sand	Boot	Naive	Sand	Boot
$\delta_{\mathrm{low}}$	$\rho_{\mathrm{low}}$	0.903	0.760	0.754	0.658	0.482	0.479	0.400	0.322	0.284
$\delta_{\mathrm{high}}$	$\rho_{\mathrm{low}}$	0.946	0.591	0.584	0.736	0.436	0.400	0.454	0.315	0.245
$\delta_{\mathrm{low}}$	$\rho_{\mathrm{high}}$	0.965	0.828	0.824	0.761	0.580	0.563	0.448	0.317	0.286
$\delta_{\mathrm{high}}$	$\rho_{\mathrm{high}}$	0.971	0.620	0.616	0.793	0.445	0.433	0.481	0.305	0.253

The improved performance of bootstrap CIs relative to sandwich CIs agrees with previous research. Robins, Rotnitzky, and Zhao (1994) recommended bootstrap in a related context, saying the sandwich estimator can suffer from finite‐sample bias. In the context of average treatment effect estimation, Austin (2016a) found the sandwich estimator underestimated the variance unless sample size is large, and bootstrap performed better. We also found the sandwich estimator tended to underestimate the variance more than did bootstrap, at least when n≥500 (see Supporting Information Section J). In the context of estimating a marginal hazard ratio, Austin (2016b) recommended bootstrap, while Mao et al. (2018) found bootstrap performed better than sandwich except when both performed badly. However, in these last two simulation studies, the data‐generating mechanisms appear not to be compatible with the analysis models, due to noncollapsibility of hazard ratios, which could adversely affect the sandwich estimator. Note that there is a sandwich variance estimator that, unlike the simple sandwich estimator used here, accounts for uncertainty in the weights. However, accounting for this uncertainty is known to reduce the variance estimate, and so would not have improved coverage in our study (Enders et al. 2018).

## 7 Discussion

Our proposed algorithm for simulating data from an MSM for a survival time outcome allows more general data‐generating mechanisms than previously proposed algorithms. However, unlike Young and Tchetgen (2014) approach, it does not allow the conditional hazard given the history of time‐dependent confounders and treatment to be directly specified, a specification which may be desirable for some simulation studies. Nor does it generate data that simultaneously satisfy multiple models, as was the goal of Young et al. (2010). So, those previous methods remain useful (but limited in the data‐generating mechanisms they allow).

Our algorithm assumes visit times are the same for all individuals and equally spaced. It can be generalized to unequally spaced visits, by rescaling the time axis between subsequent visits, provided that visit times remain the same for all individuals. In Supporting Information Section K, we adapt our algorithm to generate data for continuous‐time MSMs (Saarela and Liu 2016; Ryalen, Stensrud, and Roysland 2019; Ryalen et al. 2020; Dong 2021), where the times at which exposure and confounders change differ between individuals. We have also assumed the risk score is a continuous variable, but in Supporting Information Section L, we describe simple modifications of our algorithm to handle a discrete risk score, such as would arise if all confounders were discrete. Throughout, we have been considering static treatment regimes, that is, regimes where treatment allocation is prespecified. The problem of simulating data for dynamic treatment regimes (Young et al. 2011; Cain et al. 2016; Emilsson et al. 2017; Garcia‐Albeniz et al. 2015; Hernan 2018; Zhang et al. 2014), that is, regimes where treatment allocation depends on information collected during the study, is discussed in Supporting Information Section M.

Finally, our algorithm uses Gaussian copulas. Non‐Gaussian copulas could be used instead, which would give a different function of $U_{H_{0}^{a_{0}}}$ on the right‐hand side of Equation (4) (and analogously at other visits). However, whereas the Gaussian copula causes $P(Y_{1}^{a_{0}}=0|X,B,L_{0})$ to be an increasing function of $U_{H_{0}^{a_{0}}}$, this is not true of all copulas. Thus, although $U_{H_{0}^{a_{0}}}$ would still represent the function of $\left(B,L_{0}\right)$ through which $Y_{1}^{a_{0}}$ depends on B and $L_{0}$ (cf. the linear predictor in a logistic regression model), it might not be interpretable as a risk quantile.

## Conflicts of Interest

The authors declare no conflicts of interest.

### Open Research Badges

This article has earned an Open Data badge for making publicly available the digitally‐shareable data necessary to reproduce the reported results. The data is available in the Supporting Information section.

This article has earned an open data badge “**Reproducible Research**” for making publicly available the code necessary to reproduce the reported results. The results reported in this article could fully be reproduced.

## Supporting information

Supporting Information

Supporting Information

## Data Availability

The data that support the findings of this study are available in the Supporting Information of this article.
