# Supplementary material for: Simulating Data From Marginal Structural Models for a Survival Time Outcome
Source: Biom J. 2024 Nov 23;66(8):e70010. doi: 10.1002/bimj.70010 (PMC11585228; doi:10.1002/bimj.70010)
Supplement: Supplementary file 2 — Supporting Information [file BIMJ-66-e70010-s001.pdf]

Supplementary Materials for ‘Simulating data from marginal structural models for a survival time outcome’

Shaun R. Seaman and Ruth H. Keogh

A Evans and Didelez’s MSM simulation study

In this appendix, we discuss the model used in Evans and Didelez’s Example 6.2 and the simulation study using this model in their Appendix F. We explain how Evans and Didelez are able to simulate data from this model and discuss the restrictiveness of the model. We then describe how our unextended algorithm in Section 3 could very easily be modified to simulate from this same model (by replacing the copula by an odds ratio), and explain why our extended algorithm in Section 4 is not required.

Evans and Didelez’s Example 6.2 comes from Young and Tchetgen Tchetgen (2014)[15] (henceforth, ‘YTT’), who consider the scenario where the failure time is discrete, there are no baseline covariates X or B, the treatment A_k is binary, there only one confounder L_k, which is binary, and

$$P(Y_{k+1} = 0 \mid \bar{L}_k, \bar{A}_k, Y_k = 1) = P(Y_{k+1} = 0 \mid L_k, A_k, A_{k-1}, Y_k = 1) = s(L_k, A_k, A_{k-1}) \tag{A1}$$

and

$$P(L_k = 1 \mid \bar{L}_{k-1}, \bar{A}_{k-1}, Y_k = 1) = P(L_k = 1 \mid A_{k-1}, Y_k = 1) = r(A_{k-1}) \tag{A2}$$

for all $k = 0, \ldots, K$ and for some functions s and r. Equation (A1) means that the (discrete-time) hazard of failure depends only on the most recent treatment and confounder values. Equation (A2) means that the conditional distribution of the time-dependent confounder depends only on the most recent treatment.

YTT show that in this scenario,

$$\frac{P(Y_{k+1}^{\bar{a}_k} = 0 \mid Y_k^{\bar{a}_{k-1}} = 1)}{P(Y_{k+1}^{\bar{0}_k} = 0 \mid Y_k^{\bar{0}_{k-1}} = 1)} = \exp(\psi_0 a_k + \psi_1 a_{k-1} + \psi_2 a_k a_{k-1}) \tag{A3}$$

with ψ_0, ψ_1 and ψ_2 given by YTT’s equations (10)–(12).

More specifically, if

$$P(Y_{k+1} = 0 \mid \bar{L}_k, \bar{A}_k, Y_k = 1) = \frac{\exp(\theta_0 + \theta_1 L_k + \theta_2 A_k + \theta_3 A_{k-1})}{1 + \exp(\theta_0 + \theta_1 L_k + \theta_2 A_k + \theta_3 A_{k-1})}, \tag{A4}$$

$$P(L_k = 1 \mid \bar{L}_{k-1}, \bar{A}_{k-1}, Y_k = 1) = \frac{\exp(\beta_1 A_{k-1})}{1 + \exp(\beta_1 A_{k-1})} \tag{A5}$$

and failure is rare, so that equation (A4) becomes

$$P(Y_{k+1} = 0 \mid \bar{L}_k, \bar{A}_k, Y_k = 1) \approx \exp(\theta_0 + \theta_1 L_k + \theta_2 A_k + \theta_3 A_{k-1}), \tag{A6}$$

then ψ_0, ψ_1 and ψ_2 are given by YTT's equations (22)–(24). YTT note that this result easily generalises to the situation where the right-hand side of equation (A5) is replaced by $\exp(\beta_0 + \beta_1 A_{k-1})/\{1 + \exp(\beta_0 + \beta_1 A_{k-1})\}$.

For the remainder of this Appendix A we shall be assuming, unless indicated otherwise, that all the assumptions stated so far in this Appendix A are satisfied.

By the consistency assumption and equation (A4), we have

$$\begin{aligned} &P(Y_{k+1}^{\bar{a}_k} = 0 \mid \bar{L}_k^{\bar{a}_{k-1}} = \bar{l}_k, \bar{A}_k = \bar{a}_k, Y_k^{\bar{a}_{k-1}} = 1) \\ &\quad = P(Y_{k+1} = 0 \mid \bar{L}_k = \bar{l}_k, \bar{A}_k = \bar{a}_k, Y_k = 1) \\ &\quad = \frac{\exp(\theta_0 + \theta_1 l_k + \theta_2 a_k + \theta_3 a_{k-1})}{1 + \exp(\theta_0 + \theta_1 l_k + \theta_2 a_k + \theta_3 a_{k-1})}. \end{aligned} \tag{A7}$$

The causal DAG (Figure 1 in our article, and see also Section 3.2 of our article) implies that

$$P(Y_{k+1}^{\bar{a}_k} = 0 \mid \bar{L}_k^{\bar{a}_{k-1}}, \bar{A}_k = \bar{a}_k, Y_k^{\bar{a}_{k-1}} = 1) = P(Y_{k+1}^{\bar{a}_k} = 0 \mid \bar{L}_k^{\bar{a}_{k-1}}, Y_k^{\bar{a}_{k-1}} = 1). \tag{A8}$$

From equations (A7) and (A8), we have

$$P(Y_{k+1}^{\bar{a}_k} = 0 \mid \bar{L}_k^{\bar{a}_{k-1}}, Y_k^{\bar{a}_{k-1}} = 1) = \frac{\exp(\theta_0 + \theta_1 L_k^{\bar{a}_{k-1}} + \theta_2 a_k + \theta_3 a_{k-1})}{1 + \exp(\theta_0 + \theta_1 L_k^{\bar{a}_{k-1}} + \theta_2 a_k + \theta_3 a_{k-1})}. \tag{A9}$$

We see from equation (A9) that the risk ranking of individuals with $Y_k^{\bar{a}_{k-1}} = 1$ and different values of $\bar{L}_k^{\bar{a}_{k-1}}$ depends only on $L_k^{\bar{a}_{k-1}}$ and whether θ_1 is positive or negative. Hence, by the definition of the risk score function[1], $h_k^{\bar{a}_k}(\bar{l}_k)$ is just l_k (if $\theta_1 > 0$) or $-l_k$ (if $\theta_1 < 0$) or some monotonically increasing function of l_k (or $-l_k$). Suppose (without loss of generality) that $h_k^{\bar{a}_k}(\bar{l}_k) = l_k$, and so $H_k^{\bar{a}_k} = L_k^{\bar{a}_{k-1}}$.

We also see from equation (A9) that the odds ratio between $1 - Y_{k+1}^{\bar{a}_k}$ and $L_k^{\bar{a}_{k-1}}$ given $Y_k^{\bar{a}_{k-1}} = 1$ is $\exp(\theta_1)$. Hence, the odds ratio between $Y_{k+1}^{\bar{a}_k}$ and $L_k^{\bar{a}_{k-1}}$ given $Y_k^{\bar{a}_{k-1}} = 1$ is $\exp(-\theta_1)$.

By equation (A5), the consistency assumption and the causal DAG, we have the CDF of $L_k^{\bar{a}_k}$ given $Y_k^{\bar{a}_k} = 1$:

$$\begin{aligned} F_{L_k^{\bar{a}_k}}(0 \mid Y_k^{\bar{a}_k} = 1) &= \frac{1}{1 + \exp(\beta_1 a_{k-1})} \\ F_{L_k^{\bar{a}_k}}(1 \mid Y_k^{\bar{a}_k} = 1) &= 1. \end{aligned} \tag{A10}$$

Also, if we know $\psi_* = \log P(Y_{k+1}^{\bar{0}_k} = 0 \mid Y_k^{\bar{0}_{k-1}} = 1)$, then it follows from equation (A3) that we know the distribution of $Y_{k+1}^{\bar{a}_k}$ given $Y_k^{\bar{a}_{k-1}} = 1$:

$$P(Y_{k+1}^{\bar{a}_k} = 0 \mid Y_k^{\bar{a}_{k-1}} = 1) = \exp(\psi_* + \psi_0 a_k + \psi_1 a_{k-1} + \psi_2 a_k a_{k-1}). \tag{A11}$$

To recap, if we know $(\psi_*, \psi_0, \psi_1, \psi_2, \beta_1, \theta_1)$, then we know: i) the (marginal) distribution of the binary variable $Y_{k+1}^{\bar{a}_k}$ given $Y_k^{\bar{a}_{k-1}} = 1$; ii) the (marginal) distribution

[1]Recall that there is no baseline variables X or B in this example.

of the binary variable $L_k^{\bar{a}_{k-1}}$ given $Y_k^{\bar{a}_{k-1}} = 1$; and iii) odds ratio between $Y_{k+1}^{\bar{a}_k}$ and $L_k^{\bar{a}_{k-1}}$ given $Y_k^{\bar{a}_{k-1}} = 1$.

The joint distribution of $(Y_{k+1}^{\bar{a}_k}, L_k^{\bar{a}_{k-1}})$ given $Y_k^{\bar{a}_{k-1}} = 1$ follows immediately from i)–iii). The conditional distribution of $Y_{k+1}^{\bar{a}_k}$ given $L_k^{\bar{a}_{k-1}}$ and $Y_k^{\bar{a}_{k-1}} = 1$ then follows immediately from this joint distribution, and it is easy to simulate $(Y_{k+1}^{\bar{a}_k}, L_k^{\bar{a}_{k-1}})$ from this conditional distribution. This is almost what Evans and Didelez do. However, it is not quite what they do, because they actually specify $(\psi_0, \psi_1, \psi_2, \beta_1, \theta_0, \theta_1)$, rather than $(\psi_*, \psi_0, \psi_1, \psi_2, \beta_1, \theta_1)$. However, it is straightforward to calculate ψ_* from θ_0 and θ_1, because

$$
\begin{aligned}
\exp(\psi_*) &= P(Y_{k+1}^{\bar{0}_k} = 0 \mid Y_k^{\bar{0}_{k-1}} = 1) \\
&= \sum_{l_k=0}^{1} P(Y_{k+1}^{\bar{0}_k} = 0 \mid L_k^{\bar{0}_{k-1}} = l_k, Y_k^{\bar{0}_{k-1}} = 1) \times P(L_k^{\bar{0}_{k-1}} = l_k \mid Y_k^{\bar{0}_{k-1}} = 1) \\
&= \frac{\exp(\theta_0)}{1+\exp(\theta_0)} \times \frac{1}{1+\exp(\beta_1 \times 0)} + \frac{\exp(\theta_0+\theta_1)}{1+\exp(\theta_0+\theta_1)} \times \frac{\exp(\beta_1 \times 0)}{1+\exp(\beta_1 \times 0)} \qquad \text{(A12)} \\
&= \frac{\exp(\theta_0)}{2\{1+\exp(\theta_0)\}} + \frac{\exp(\theta_0+\theta_1)}{2\{1+\exp(\theta_0+\theta_1)\}}.
\end{aligned}
$$

Note that line (A12) follows from equations (A9) and (A10).

Although this approach of Evans and Didelez works when there are no baseline covariates (X, B), L_k is a single binary variable, failure is rare and equations (A3)–(A5) hold, Evans and Didelez do not say what they would do if L_k were continuous and/or a vector, or when equations (A3)–(A5) do not hold. For example, YTT also consider the corresponding scenario where, instead of being a single binary variable, L_k is a single continuous variable with $L_k \mid \bar{L}_{k-1}, \bar{A}_{k-1}, Y_k = 1 \sim \text{Normal}(\beta_1 A_{k-1}, \sigma^2)$.

Our approach of using a copula to describe the association between $Y_{k+1}^{\bar{a}_k}$ and $L_k^{\bar{a}_{k-1}}$ given $Y_k^{\bar{a}_{k-1}} = 1$ would not work in the scenario of a single binary L_k, because we assume that $H_k^{\bar{a}_k}$ is a continuous random variable, whereas here $H_k^{\bar{a}_k} = L_k$ is binary. However, if we take our method and replace the copula by an odds ratio parameter, then it does exactly what Evans and Didelez have done. This is what we label as 'Option 2' in our Appendix L. Alternatively, we could continue to use a copula but with the modification described as 'Option 1' in Appendix L.

Finally, we remark that in the scenario that Evans and Didelez consider, $H_k^{\bar{a}_k} = L_k^{\bar{a}_k}$ and $F_{H_k^{\bar{a}_k}}(h \mid Y_k^{\bar{a}_k} = 1)$ is a known function (see equation (A10)). This remains true in the more general scenario where equation (A1) holds and L_k can be discrete or continuous and can be scalar or vector but obeys

$$p(L_k \mid \bar{L}_{k-1}, \bar{A}_{k-1}, Y_k = 1) = p(L_k \mid \bar{A}_{k-1}, Y_k = 1).$$

Recall that when $F_{H_k^{\bar{a}_k}}(h \mid Y_k^{\bar{a}_k} = 1)$ is a known function, there is no need to estimate it using the 'extended algorithm' described in Section 4 of our article.

B Introduction to Gaussian copulas

Let V_1 and V_2 be two random variables and let $F_{V_1}(v_1)$ and $F_{V_2}(v_2)$ denote their marginal CDFs. In the context of this article, V_1 and V_2 represent $U_{Y_{k+1}^{\bar{a}_k}}$ and $H_k^{\bar{a}_k}$, respectively, and all distributions are conditional on X and $Y_k^{\bar{a}_{k-1}} = 1$. Let $U_1 = F_{V_1}(V_1)$ and $U_2 = F_{V_2}(V_2)$. By a general property of CDFs, the marginal distributions of U_1 and U_2 are both Uniform$(0, 1)$. The copula of (V_1, V_2) is defined as the joint CDF of (U_1, U_2):

$$C(u_1, u_2) = P(U_1 \leq u_1, U_2 \leq u_2) \qquad (0 \leq u_1 \leq 1,\ 0 \leq u_2 \leq 1). \tag{A13}$$

Various parametric forms can be assumed for this copula (see, for example, the appendices of [1, 13] for examples). A commonly-used form is the Gaussian copula with correlation parameter ρ $(-1 \leq \rho \leq 1)$:

$$C(u_1, u_1; \rho) = \frac{1}{\sqrt{1-\rho^2}} \exp\left\{-\frac{\rho^2(u_1^2 + u_2^2) - 2\rho u_1 u_2}{2(1-\rho^2)}\right\}. \tag{A14}$$

Postulating this copula is equivalent to assuming that $(\Phi^{-1}(U_1), \Phi^{-1}(U_2))$ has a bivariate normal distribution with marginal means 1, marginal variances 0 and correlation ρ. Here, $\Phi(.)$ denotes the CDF of a standard (univariate) normal distribution, and $\Phi^{-1}(.)$ is its inverse.

The conditional CDF of U_1 given $U_2 = u_2$ implied by equation (A13) is[13]

$$F_{U_1}(u_1 \mid u_2) = \frac{\partial}{\partial u_1} C(u_1, u_2).$$

In the case of the Gaussian copula, this is

$$F_{U_1}(u_1 \mid u_2) = \Phi\left(\frac{\Phi^{-1}(u_1) - \rho\Phi^{-1}(u_2)}{\sqrt{1-\rho^2}}\right). \tag{A15}$$

Equation (A15) is equivalent to $\Phi^{-1}(U_1) \mid U_2 = u_2 \sim \text{Normal}(\rho\Phi^{-1}(u_2), 1-\rho^2)$, i.e. the conditional normal distribution implied by the bivariate normal distribution of $(\Phi^{-1}(U_1), \Phi^{-1}(U_2))$.

More generally, expressions for the conditional CDF $F_{U_1}(u_1 \mid u_2)$ of U_1 given $U_2 = u_2$ are also available for many other parametric copula forms[1, 13].

V_2 can be sampled from its conditional distribution given V_2 as follows.

1. Calculate $U_2 = F_{V_2}(V_2)$.
2. Generate $Q \sim \text{Uniform}(0, 1)$.
3. Calculate $U_1 = F_{U_1}^{-1}(Q \mid U_2)$.
4. Calculate $V_1 = F_{V_1}^{-1}(U_1)$.

In the case of the Gaussian copula, these steps are equivalent to the following steps.

1. Calculate $U_2 = F_{V_2}(V_2)$.
2. Calculate $Z_2 = \Phi^{-1}(U_2)$.
3. Generate $Z_1 \sim \text{Normal}(\rho Z_2, 1-\rho^2)$.
4. Calculate $U_1 = \Phi(Z_1)$.
5. Calculate $V_1 = F_{V_1}^{-1}(U_1)$.

That is how the procedure was described in the article. In the article, V_1 represents $U_{Y_{k+1}^{\bar{a}_k}}$, which is distributed Uniform(0, 1) (given X and $Y_k^{\bar{a}_{k-1}} = 1$), and so $V_1 = F_{V_1}^{-1}(U_1) = U_1$.

C Proof of equation in Section 3.2

By the consistency assumption,

$$P(Y_2^{\bar{a}_1} = 0 \mid X, B, L_0, \bar{A}_1 = \bar{a}_1, Y_1 = 1, L_1)$$
$$= P(Y_2^{\bar{a}_1} = 0 \mid X, B, L_0, A_0 = a_0, A_1^{a_0} = a_1, Y_1^{a_0} = 1, L_1^{a_0}). \quad \text{(A16)}$$

Figure A1 shows the Single World Intervention Graph (SWIG)[9] that corresponds to the causal DAG of Figure 1 after intervening to set $(A_0, A_1) = (a_0, a_1)$. By applying d-separation (see Section 3.5.2 of [9]) to this SWIG, it can be seen that

$$Y_2^{\bar{a}_1} \perp\!\!\!\perp (A_0, A_1^{a_0}) \mid X, B, L_0, Y_1^{a_0} = 1, L_1^{a_0}. \quad \text{(A17)}$$

It follows from equation (A16) and expression (A17) that

$$P(Y_2^{\bar{a}_1} = 0 \mid X, B, L_0, \bar{A}_1 = a_1, Y_1 = 1, L_1) = P(Y_2^{\bar{a}_1} = 0 \mid X, B, L_0, Y_1^{a_0} = 1, L_1^{a_0}).$$

D Estimating CDF of risk score when $(X, \bar{A}_K)$ can take few values

As mentioned in Section 4, when the number of possible values of $(X, \bar{A}_K)$ is finite and fairly small, the CDF $F_{H_k^{\bar{a}_k}}(h \mid x, Y_k^{\bar{a}_{k-1}} = 1)$ of the risk score can be estimated separately for each of these possible values by a Monte Carlo procedure prior to applying the algorithm in Section 3.3. Here we describe that Monte Carlo procedure.

Let m be a large number, e.g. $m = 10^6$. For each possible value $(x, \bar{a}_K)$ of $(X, \bar{A}_K)$, follow these steps.

1. For $j = 1, \ldots, m$, sample B_j from $p(B_j \mid X_j = x)$. Set $k = 0$.
2. For $j = 1, \ldots, m$, sample L_{kj} from $p(L_{kj} \mid X_j = x, B_j, \bar{L}_{k-1,j}, \bar{A}_{k-1,j} = \bar{a}_{k-1}, Y_{kj} = 1)$.

3. For $j = 1, \ldots, m$, calculate $H_{kj} = H_k^{\bar{a}_k}(x, B_j, \bar{L}_{kj})$. Let $\hat{F}_{H_k^{\bar{a}_k}}(h \mid x, Y_k^{\bar{a}_{k-1}} = 1)$ denote the empirical distribution of $\{H_{k1}, \ldots, H_{km}\}$.

4. If $k = K$, stop.

5. Let R_{kj} denote the rank of H_{kj} among the set $\{H_{k1}, \ldots, H_{km}\}$.

6. Generate m independent Uniform$(0, 1)$ random variables, $W_{k1}, \ldots, W_{km}$. For $j = 1, \ldots, m$, calculate $U_{H_{kj}} = (R_{kj} - W_{kj})/m$.

7. For $j = 1, \ldots, m$, calculate $Z_{H_k^{\bar{a}_k},j} = \Phi^{-1}(U_{H_k^{\bar{a}_k},j})$.

8. For $j = 1, \ldots, m$, Sample $Z_{Y_{k+1}^{\bar{a}_k},j} \sim \text{Normal}(\rho_k Z_{H_k^{\bar{a}_k},j}, 1 - \rho_k^2)$ and calculate $U_{Y_{k+1}^{\bar{a}_k},j} = \Phi(Z_{Y_{k+1}^{\bar{a}_k},j})$.

9. For $j = 1, \ldots, m$, set $Y_{k+1,j} = 0$ if $U_{Y_{k+1},j} < g_{k+1}(\bar{a}_{k-1}, x; \beta)$ and set $Y_{k+1,j} = 1$ otherwise.

10. Replace each individual j that has $Y_{k+1,j} = 0$ with a randomly chosen individual j^* $(2 \leq j^* \leq m)$ that has $Y_{k+1,j^*} = 1$. That is, randomly generate j^* and then set $B_j = B_{j^*}$, $\bar{L}_{kj} = \bar{L}_{kj^*}$ and $Y_{k+1,j} = 1$.

11. Let $k = k + 1$ and return to step 2.

Now $\hat{F}_{H_k^{\bar{a}_k}}(h \mid x, Y_k^{\bar{a}_{k-1}} = 1)$ (calculated at step 3) is an estimate of $F_{H_k^{\bar{a}_k}}(h \mid x, Y_k^{\bar{a}_{k-1}} = 1)$ that can be used in the algorithm of Section 3.3.

In practice, one might store only a fixed number of quantiles of this empirical distribution function and then interpolate between these when using the algorithm of Section 3.3. For example, these could be the $(0.00001, 0.00002, \ldots 0.0001)$th, the $(0.0002, 0.0003, \ldots 0.1)$th and the $(0.101, 0.102, 0.103, \ldots, 0.5)$th quantiles (a total of $10 + 999 + 400 = 1409$ quantiles) and the corresponding 1408 quantiles between 0.501 and 0.99999.

E Additional step for algorithm of Section 4

As explained at the end of Section 4, the number of distinct values of $\{I_2, \ldots, I_m\}$ could eventually be small, which may cause the performance of the algorithm to deteriorate. To deal with the issue, we modify step 12 and add a new step 13. The new steps 12 and 13 are as follows.

12. If the number of unique values in the set $\{I_2, \ldots, I_m\}$ is greater than some threshold, e.g. $m/10$, then let $k = k + 1$ and return to step 3.

13. Let k^* denote the current value of k, i.e. the visit reached just before entering this step. Set $k = 0$, $m = 100000$ and $X_j = X_1$ $(j = 2, \ldots, m)$,

    (a) Repeat step 2 for $j = 2, \ldots, m$ (i.e. for the matches only).

(b) Repeat step 3, but only include $j = 1$ if $k > k^*$.

(c) Repeat step 4 if $k > k^*$.

(d) Repeat steps 5–8.

(e) Repeat step 9, but only include $j = 1$ if $k > k^*$.

(f) Repeat steps 10 and 11.

(g) Let $k = k + 1$ and return to step 12b.

Note that the reason for omitting $j = 1$ at steps 13b and 13e when $k \leq k^*$ and for step 13c not repeating step 4 when $k \leq k^*$ is that the variables $L_0, A_0, Y_0, \ldots, L_{k^*}, A_{k^*}, Y_{k^*+1}$ have already been generated for the sampled individual $j = 1$. This individual has already managed to survive to time k^*+1 and should not be exposed to the 'double jeopardy' of being tested twice for failure at any time before time $k^* + 2$.

F Empirical study

In this section, we demonstrate that the extended algorithm does indeed generate data compatible with a pre-specified MSM.

We let $K = 9$ and consider a binary treatment A_k. We choose to have two independent baseline covariates $X = (X_1, X_2)^\top$ in the MSM, and two baseline variables $B = (B_1, B_2)^\top$ that are not in the MSM, and assume that B_1 and B_2 are conditionally independent given X. Further, $L_k = (L_{k1}, L_{k2})^\top$ are two time-dependent variables that are conditionally independent given $(X, B, \bar{L}_{k-1}, \bar{A}_{k-1})$ and $Y_k = 1$ ($k = 0, \ldots, 9$). We specify that

$$
\begin{aligned}
X_1 &\sim \text{Normal}(0, 1) \\
X_2 &\sim \text{Bernoulli}(0.5) \\
B_1 \mid X &\sim \text{Normal}(-0.2 + 0.4X_2, 1) \\
B_2 \mid X &\sim \text{Normal}(0.2X_1, 1) \\
L_{01} \mid X, B &\sim \text{Normal}(0.2X_1, 1) \\
L_{02} \mid X, B &\sim \text{Bernoulli}(\text{expit}(-0.2 + 0.4X_2))
\end{aligned}
$$

and

$$
\begin{aligned}
L_{k1} \mid X, B, \bar{L}_{k-1}, \bar{A}_{k-1}, Y_k = 1 &\sim \text{Normal}(0.3 + 0.4B_2 + 0.7L_{k-1,1} - 0.6A_{k-1}, 1) \\
L_{k2} \mid X, B, \bar{L}_{k-1}, \bar{A}_{k-1}, Y_k = 1 &\sim \text{Bernoulli}(\text{expit}(-0.2 + 0.4B_2 + L_{k-1,2} - 0.6A_{k-1}))
\end{aligned}
$$

for $k = 1, \ldots, 9$. We want to simulate data compatible with the following marginal structural logistic model:

$$
P(Y_{k+1}^{\bar{a}_k} = 0 \mid X, Y_k^{\bar{a}_k} = 1) = \text{expit}(\beta_{k0} + \beta_{k1}X_1 + \beta_{k2}X_2 + \beta_{k3}a_k) \qquad (k = 0, \ldots, 9).
$$

We choose the risk score function to be $h_k^{\bar{a}_k}(x, b, \bar{l}_k) = 0.3b_1 + 0.5b_2 + l_{k1} + l_{k2}$ and the correlation parameter of the Gaussian copula to be $\rho_k = -0.9$. This means that,

under the interventional regime (i.e. when $\bar{A}_K$ is set equal to $\bar{a}_K$), an individual with large values of B_1, B_2, L_{k1} and L_{k2} is more likely to fail at time $k+1$ than is the average individual with the same value of X.

For the treatment, we specify that

$$P(A_k = 1 \mid X, B, \bar{L}_k, \bar{A}_{k-1}, Y_k = 1) = \\ \text{expit}(-1 + 0.2X_1 + 0.3X_2 + 0.2B_1 + 0.6L_{k1} + 0.6L_{k2} + A_{k-1}). \quad \text{(A18)}$$

It can be seen that X_1, X_2, B_1, L_{k1} and L_{k2} influence both A_k and $Y_{k+1}^{\bar{a}_k}$, and are hence confounders. The variable B_2 is a common cause of L_k and $Y_{k+1}^{\bar{a}_k}$ but not a confounder, because it does not (directly) influence A_k. We shall regard B_2 as an unobserved variable; the algorithm will generate values for B_2 but we shall discard these data.

The true values of the causal log odds ratio parameters in the MSM were chosen to be $(\beta_{k1}, \beta_{k2}, \beta_{k3}) = (0.5, 0.5, -1)$. Three sets of values were considered for the baseline log odds parameters: $\beta_{k0} = -4.1$, $\beta_{k0} = -2.5$ and $\beta_{k0} = -1.2$. When $\beta_{k0} = -4.1$, approximately 90% of individuals survive to time $K + 1 = 10$, i.e. $P(Y_{10} = 1) \approx 0.9$. Approximately 50% survive when $\beta_{k0} = -2.5$, and approximately 10% survive when $\beta_{k0} = -1.2$.

All the quantities needed to determine the data-generating mechanism have now been specified. For each of the three values of β_{k0}, we used the algorithm in Section 4 to generate a sample of size $n = 10^6$ individuals.

To each of the three samples, we fitted the following MSM twice, once without weighting the data and once using inverse probability of treatment (stabilised) weights:

$$P(Y_{k+1}^{\bar{a}_k} = 0 \mid X, Y_k^{\bar{a}_k} = 1) = \\ \text{expit}(\beta_{k0} + \beta_1 X_1 + \beta_2 X_2 + \beta_3 a_k + \beta_4 X_1 k + \beta_5 X_2 k + \beta_6 a_k k) \\ (k = 0, \ldots, 9).$$

Obviously, the true values of the parameters are $\beta_1 = 0.5$, $\beta_2 = 0.5$, $\beta_3 = -1$ and $\beta_4 = \beta_5 = \beta_6 = 0$. The weights at visit k were calculated in the standard way[8] as

$$\prod_{j=0}^{k} \frac{\hat{p}(A_j \mid X, \bar{A}_{j-1}, Y_j = 1)}{\hat{p}(A_j \mid X, B_1, \bar{L}_j, \bar{A}_{j-1}, Y_j = 1)}, \quad \text{(A19)}$$

with the terms in the denominator of expression (A19) calculated by fitting the correctly specified logistic regression model for A_j given $X, B_1, \bar{L}_j, \bar{A}_{j-1}$ and $Y_j = 1$ (see equation (A18)) to the sample. The terms in the numerator were calculated by fitting the corresponding (misspecified) logistic regression model that omits B_1 and $\bar{L}_j$.

Table A1 shows the point estimates obtained when $\beta_{k0} = -2.5$, i.e. when 50% of individuals fail during follow-up. Also shown are standard errors (SEs) of these estimates, calculated using either the Fisher information (for the point estimates obtained without weighting) or the sandwich variance estimator (for the point

estimates obtained using IPTW). As expected, the unweighted point estimates are biased, particularly that of β_3. The point estimates obtained using IPTW, on the other hand, are very close to the true values of the parameters. In particular, all estimates are within two SEs of the true parameter values. Analogous results for 10% failure and 90% failure are given in Tables A2 and A3. In both cases, all the estimates are very close to the true parameter values. These results verify that our algorithm is generating data compatible with the chosen MSM.

G Sensitivity of simulated failure times to the number of matches

In Appendix F, 4999 matches were used to estimate the CDF of the risk score. Since the data for these matches are generated randomly, a different set of 4999 matches, or a smaller number of matches, would yield a different estimate of this CDF. This different estimate might then affect the failure times generated for the n sampled individuals. To assess how sensitive these n failure times are to this estimation uncertainty, we carried out the following investigation.

Using the same data-generating mechanism as in the empirical study, we generated a value of $(B, X, \bar{L}_{10}, \bar{A}_{10})$ for each of $n = 100000$ (one hundred thousand) individuals. Just as in the extended algorithm of Section 4, this was done by sampling L_k from $p(L_k \mid X, B, \bar{L}_{k-1}, \bar{A}_{k-1}, Y_k = 1)$ and A_k from $p(A_k \mid X, B, \bar{L}_k, \bar{A}_{k-1}, Y_k = 1)$ (for $k = 0, \ldots, 10$). We also simulated values of $W_{k1} \sim \text{Uniform}(0, 1)$ ($k = 1, \ldots, 10$) for each of the 100000 individuals. These W_{k1}'s were to be used in step 6 of the extended algorithm. In addition, we simulated values of $S_{k1} \sim \text{Normal}(0, 1 - \rho_k^2)$ (for $k = 1, \ldots, 10$) for each of the 100000 individuals. These were to be used in step 8 of the extended algorithm (see later for how they were used). These 100000 values of $(B, X, \bar{L}_{10}, \bar{A}_{10}, W_{k1}, \ldots, W_{k,10}, S_{k1}, \ldots, S_{k,10})$ were generated only once and then used throughout this investigation.

Next, for each of five values of m (see Table A4), we used a slightly modified version of the extended algorithm to generate a failure time for each of the $n = 100000$ sampled individuals. The reason for this modification was to ensure that the only reason for changes in the generated failure times of the $n = 100000$ sampled individuals was the data generated for the matches. The modification was that:

- Step 3 was only applied to $j = 2, \ldots, m$, i.e. the matches, because we had already generated $\bar{L}_{10,1}$.
- Step 4 was omitted (except to set a_k equal to A_{k1}), because we had already generated $\bar{A}_{10,1}$.
- In step 6, we did not generate W_{k1}, because we had already generated it (see above). Note that this ensures that when H_{k1} is fixed, the only randomness in $U_{H_k^{\bar{a}_k},1}$ comes from the H_{kj} values ($j = 2, \ldots, m$) of the matches.

- In step 8, we set $Z_{Y^{\bar{a}_k}_{k+1},1} = \rho_k Z_{H^{\bar{a}_k}_k,1} + S_{k1}$. Again, note that this ensures that when H_{k1} is fixed, the only randomness in $Z_{Y^{\bar{a}_k}_{k+1},1}$ comes from the H_{kj} values ($j = 2, \ldots, m$) of the matches.

Using each of the five m values (Table A4) in turn, we applied the extended algorithm with these modifications. We then repeated this procedure using a different random number seed.

We shall refer to the scenarios where $\beta_{k0} = -4.1$ (and so 90% of individuals survive), $\beta_{k0} = -2.5$ (50% survive) and $\beta_{k0} = -1.2$ (10% survive) as the 'low-risk', 'medium-risk' and 'high-risk' scenarios, respectively. Table A5 shows, for each scenario, the percentage of the $n = 100000$ failure times that were unchanged when we used a different value of m or when we used the same value of m but a different random number seed.

When 4999 matches ($m = 5000$) were used, between 97% (in the medium and high-risk scenarios) and 99% (in the low-risk scenario) of failure times were unchanged when the random number seed was changed. When 999 matches ($m = 1000$) were used, between 94% (high-risk scenario) and 98% (low-risk scenario) of failure times were the same as they had been when 4999 matches were used. When 499 matches were used, between 92% and 97% of failure times were the same as they had been when 4999 matches were used. When 99 matches were used, between 84% and 93% were the same. Obviously, we would never consider using only nine matches in practice, but, nonetheless, it is interesting to observe that when $m = 10$, up to 37% of failure times were different, confirming that $m = 10$ is a poor choice.

The results presented above were obtained using $\rho = -0.9$ as the correlation parameter of the Gaussian copula. When ρ is smaller (i.e. closer to zero) than -0.9, the dependence of $Z_{Y^{\bar{a}_k}_{k+1},1}$ (in Step 8 of the extended algorithm) on the data generated for the matches will be reduced, suggesting that a smaller m could be used in this case. Table A6 shows results obtained when $\rho = -0.5$. Here we see that when 499 matches were used, over 95% of failure times were the same as they had been when 4999 matches were used.

On the basis of these results, we recommend using at least $m = 1000$ when the marginal risk of failure is moderate/high and ρ is large. However, if computation time is an issue, $m = 500$ may suffice when the marginal risk is low and/or ρ is small. In our empirical study, the times taken to generate the data for $n = 1000000$ (one million) sampled individuals using $m = 5000$ were 3.5, 2.9 and 2.1 hours for the low-risk, medium-risk and high-risk scenarios, respectively. This was using a single processor of a Dell Latitude 5520 laptop with 4.7GHz 4-core Intel i7 processor. These $n = 1000000$ sampled individuals would suffice for, for example, a simulation study involving 1000 data sets each of 1000 individuals.

H Continuous failure time

In this appendix, we extend the algorithms described in our article, so that they simulate data for a continuous-time failure model. Such models include Cox and additive hazards MSMs.

By definition, $Y_k^{\bar{a}_k} = I(T^{\bar{a}_k} > k)$ $(k = 1, \ldots, K+1)$. The conditional CDF of $T^{\bar{a}_k}$ given X and $T^{\bar{a}_k} \geq k$ is, when $k < t \leq k+1$,

$$F_{T^{\bar{a}_k}}(t \mid X = x, T^{\bar{a}_k} \geq k) = 1 - \exp\left\{ - \int_k^t \lambda^{\bar{a}_k}(s \mid X = x;\ \beta)\, ds \right\}. \qquad \text{(A20)}$$

This implies that

$$\begin{aligned} g_{k+1}(\bar{a}_k, x; \beta) &= P(Y_{k+1}^{\bar{a}_k} = 0 \mid X = x, Y_k^{\bar{a}_k} = 1) \\ &= F_{T^{\bar{a}_k}}(k+1 \mid X = x, T^{\bar{a}_k} \geq k) \\ &= 1 - \exp\left\{ - \int_k^{k+1} \lambda^{\bar{a}_k}(s \mid X = x;\ \beta)\, ds \right\}. \qquad \text{(A21)} \end{aligned}$$

For simplicity, we shall assume that $\lambda^{\bar{a}_k}(t \mid X = x; \beta)$ does not change over the time interval $t \in (k, k+1]$. This would be true, for example, for a Cox MSM with baseline hazard $\lambda^{\bar{0}_K}(t \mid X = 0)$ that is constant over $t \in (k, k+1]$.

In Sections 3 and 4, we assumed $P(Y_{k+1}^{\bar{a}_k} = 0 \mid X = x, B = b, \bar{L}_k^{\bar{a}_{k-1}} = \bar{l}_k, Y_k^{\bar{a}_{k-1}} = 1)$ depends on x, b and $\bar{l}_k$ only through x and the risk score function $h_k^{\bar{a}_k}(x, b, \bar{l}_k)$. Now we assume this is also true of $\lambda^{\bar{a}_k}(t \mid x, b, \bar{l}_k)$, the potential hazard at time t $(k < t \leq k+1)$ given $X = x$, $B = b$ and $\bar{L}_k^{\bar{a}_{k-1}} = \bar{l}_k$ when we intervene to set $\bar{A}_k = \bar{a}_k$. It then follows from equations (A20) and (A21) that, when $0 \leq u \leq g_{k+1}(\bar{a}_k, x; \beta)$,

$$F^{-1}_{T^{\bar{a}_k}}(k + u \mid X = x, Y_k^{\bar{a}_k} = 1) \quad = \quad \frac{\log(1-u)}{\log\{1 - g_{k+1}(\bar{a}_k, x; \beta)\}}.$$

This means we can use the algorithm in Section 3.3 with $g_k(\bar{a}_{k-1}, X; \beta)$ defined by equation (A21) and the following additional step:

9. If $Y_{k+1} = 0$, calculate $T = k + \log(1 - U_{Y_{k+1}^{\bar{a}_k}}) / \log\{1 - g_{k+1}(\bar{a}_k, X; \beta)\}$.

This step means that when the sampled individual fails between times k and $k+1$, its continuous failure time is calculated from $U_{Y_{k+1}^{\bar{a}_k}}$. If one were instead using the extended algorithm of Section 4, its step 10 would be modified to:

10. If $Y_{k+1,1} = 0$, calculate $T_1 = k + \log(1 - U_{Y_{k+1,1}^{\bar{a}_k}}) / \log\{1 - g_{k+1}(\bar{a}_k, X_1; \beta)\}$ and then stop. If $k = K$, stop.

Note that for the Cox MSM $\lambda^{\bar{a}_k}(s \mid X = x;\ \beta) = \lambda_{k0} \exp\{\beta_k^\top q_k(\bar{a}_k, x)\}$ with constant baseline hazard λ_{k0} between times k and $k+1$, equation (A21) becomes

$$g_{k+1}(\bar{a}_k, x; \beta) = 1 - \exp\left[- \exp\left\{ \log \lambda_{k0} + \beta_k^\top q_k(\bar{a}_k, x) \right\} \right],$$

which corresponds to a MSM for a discrete failure time with complementary log log link function.

I Stabilised IPT weights in simulation study

When using IPTW in the simulation study, we calculated the (stabilised) weights at visit k as

$$\prod_{j=0}^{k} \frac{\hat{p}(A_j \mid X, \bar{A}_{j-1}, Y_j = 1)}{\hat{p}(A_j \mid X, B_1, \bar{L}_j, \bar{A}_{j-1}, Y_j = 1)}, \tag{A22}$$

with the terms in the denominator of expression (A22) calculated by fitting a correctly specified logistic regression model to the data pooled across all visits, allowing a separate intercept for each visit but assuming other coefficients to be the same at all visits. The terms in the numerator of (A22) were calculated by fitting the corresponding (misspecified) model that omits B_1 and $\bar{L}_j$.

J Variance estimates in simulation study

For the simulation study reported in Section 6, Table A7 shows the empirical SE of the IPTW estimators of $\beta_1, \ldots, \beta_5$, and the means (over the 1000 simulations) of the corresponding estimated SEs calculated using the sandwich variance estimator or bootstrap variance estimator. Note that when $n = 250$, the Monte Carlo SEs of the mean estimated SE calculated from the bootstrap variance estimator can be large relative to those of the mean estimated SE calculated from the sandwich variance estimator. The maximum Monte Carlo SE for the sandwich method is only 0.012, whereas that for the bootstrap method is 0.057. This reflects very poor performance of the bootstrap variance estimator in a small number of simulated data sets, a performance which causes considerable bias in the SE estimator for some of the parameter estimators. Despite this occasional poor performance, the coverage of bootstrap CIs is still better than that of sandwich CIs (see Table 2).

K Simulating data for a continuous-time MSM

In this appendix, we present an algorithm for generating data consistent with a MSM for a survival time outcome when the confounders and treatment can change value at different times and the times at which they change are different for different individuals. This algorithm could be used to simulate data for the continuous-time MSMs considered by Saarela et al. (2016)[12], Ryalen et al. (2019, 2020)[11, 10] and Dong (2021)[3], but can also be used to simulate data for more general continuous-time MSMs. Unlike the data-generation methods used for simulation studies by the forementioned authors, our algorithm allows the MSM of interest and the values of its parameters to be specified directly. Saarela et al. (2016) ensure that their chosen MSM is approximately correctly specified by assuming that the hazard of a change in the confounder does not depend on past or current treatment and that failure is rare. Ryalen et al. (2019) ensure that their chosen MSM is correctly specified by assuming that the hazard of a change in the exposure given the history of the exposure and confounder depends only on the current value of the confounder and,

likewise, the hazard of a change in the confounder depends only on the current exposure value. Dong (2021) similarly makes restrictive assumptions about the data-generating mechanism; it is also not clear why the assumed MSM would be correctly specified for her data-generating mechanism. Our algorithm allows a much richer choice of data-generating mechanism than those used by Saarela et al. (2016), Ryalen et al. (2019) and Dong (2021).

Note that, although in principle the algorithm that we now describe places no restriction on the number of times that an individual's treatment and confounder values change over time, this algorithm could be computationally expensive if such changes were frequent.

Let τ denote an administrative censoring time; we shall not generate data beyond time τ. Let X denote baseline variables that will be included as covariates in the MSM of interest. Let B denote baseline variables that are not included as covariates in the MSM. Just as in Section 2, B can include baseline confounders, common causes of the confounder and failure processes that are not confounders, and instrumental variables. Denote an individual's treatment and time-dependent confounder processes as $\big(A(t) : t \geq 0\big)$ and $\big(L(t) : t \geq 0\big)$, respectively. We assume that $A(t)$ is discrete. Let $\big(N^A(t) : t \geq 0\big)$ and $\big(N^L(t) : t \geq 0\big)$ denote the counting processes that jump when, respectively, $A(t)$ and $L(t)$ change value, i.e. $dN^A(t) = I\{A(t) \neq \lim_{s \to t\text{-}} A(s)\}$ and $dN^L(t) = I\{L(t) \neq \lim_{s \to t\text{-}} L(s)\}$. Let T denote the individual's failure time, and let $\big(N^T(t) : t \geq 0\big)$ denote the counting process that jumps when the individual fails, i.e. $N^T(t) = I(T \leq t)$. Let $\bar{A}(t) = \big\{A(s) : 0 \leq s \leq t\big\}$, $\bar{L}(t) = \big\{L(s) : 0 \leq s \leq t\big\}$ and $\bar{N}^T(t) = \big\{N^T(s) : 0 \leq s \leq t\big\}$. Define $\bar{A}(t\text{-}) = \big\{A(s) : 0 \leq s < t\big\}$ and similarly $\bar{L}(t\text{-})$ and $\bar{N}^T(t\text{-})$.[2]

Let $\mathcal{F}_t = \sigma\{X, B, \bar{A}(t), \bar{L}(t), \bar{N}^T(t)\}$ denote the sigma algebra generated by X, B, $\bar{A}(t)$, $\bar{L}(t)$ and $\bar{N}^T(t)$. So, $\{\mathcal{F}_t : t \geq 0\}$ is the filtration generated by X, B and the processes $\big(A(t) : t \geq 0\big)$, $\big(L(t) : t \geq 0\big)$ and $\big(N^T(t) : t \geq 0\big)$.

Let $\lambda^A(t \mid \mathcal{F}_{t\text{-}})$ denote the intensity at time t for the counting process $\big(N^A(t) : t \geq 0\big)$ with respect to filtration $\mathcal{F}_t$, and write this intensity in the form $\lambda^A(t \mid \mathcal{F}_{t\text{-}}) = \{1 - N^T(t\text{-})\} \times \alpha^A\{t \mid X, B, \bar{A}(t\text{-}), \bar{L}(t\text{-})\}$. Let $p^A\{a(t) \mid X, B, \bar{A}(t\text{-}), \bar{L}(t\text{-}), dN^A(t) = 1\}$ denote the probability mass function of $A(t)$ given the history and that a change in treatment occurs at time t. Similarly, let $\lambda^L(t \mid \mathcal{F}_{t\text{-}}) = \{1 - N^T(t\text{-})\} \times \alpha^L\{t \mid X, B, \bar{A}(t\text{-}), \bar{L}(t\text{-})\}$ denote the intensity at time t for $\big(N^L(t) : t \geq 0\big)$ with respect to $\mathcal{F}_t$, and let $p^L\{l(t) \mid X, B, \bar{A}(t\text{-}), \bar{L}(t\text{-}), dN^L(t) = 1\}$ denote the probability mass/density function of $L(t)$ given the history and that a change in confounders occurs at time t. Let $\lambda^T(t \mid \mathcal{F}_{t\text{-}}) = \{1 - N^T(t\text{-})\} \times \alpha^T\{t \mid X, B, \bar{A}(t\text{-}), \bar{L}(t\text{-})\}$ denote the intensity at time t for the process $\big(N^T(t) : t \geq 0\big)$ with respect to $\mathcal{F}_t$. So, $\alpha^T\{t \mid X, B, \bar{A}(t\text{-}), \bar{L}(t\text{-})\}$ is the hazard of failure at time t given X, B and the

[2] Readers familiar with Ryalen et al. (2019, 2020) may notice that Ryalen et al. describe the confounder process in a different way from that used in this paragraph. In their formulation, rather than having one counting process that describes the times at which the value of the vector of confounders changes, they would have one counting process for each possible value of the (assumed discrete) confounder vector. In fact, they concentrate on the specific scenario where there is only one confounder, whose value is initially 0, may change later to 1, and cannot subsequently change back from 1 to 0.

exposure and confounder histories.

Let $\mathcal{F}_t^* = \sigma\{X, \bar{A}(t), \bar{N}^T(t)\}$ denote the sigma algebra generated by X, $\bar{A}(t)$ and $\bar{N}^T(t)$. So, $\{\mathcal{F}_t^* : t \geq 0\}$ is the filtration generated by X and processes $\big(A(t) : t \geq 0\big)$ and $\big(N^T(t) : t \geq 0\big)$. Clearly, $\mathcal{F}_t^*$ is a sub-sigma algebra of $\mathcal{F}_t$. Let $\lambda^{*T}(t \mid \mathcal{F}_{t\text{-}}^*) = \{1 - N^T(t\text{-})\} \times \alpha^{*T}\{t \mid X, \bar{A}(t\text{-})\}$ denote the intensity of the process $\big(N^T(t) : t \geq 0\big)$ with respect to filtration $\mathcal{F}_t^*$. So, $\alpha^{*T}\{t \mid X, \bar{A}(t\text{-})\}$ is the hazard of failure given X and the exposure history.

Analogously to Ryalen et al. (2019) and Dong (2021, page 30), we make the local independence assumption that intervening to set $\bar{A}(\tau)$ equal to some value $\bar{a} = \bar{a}(\tau)$ does not change the intensity function $\lambda^L(t \mid \mathcal{F}_{t\text{-}})$, the probability mass/density function $p^L\{l(t) \mid X, B, \bar{A}(t\text{-}), \bar{L}(t\text{-}), dN^L(t) = 1\}$ or the intensity function $\lambda^T(t \mid \mathcal{F}_{t\text{-}})$. Hence, in particular, $\alpha^T\{t \mid X = x, B = b, \bar{A}(t\text{-}) = \bar{a}(t\text{-}), \bar{L}(t\text{-}) = \bar{l}(t\text{-})\}$ is equal to $\alpha^{T,\bar{a}}\{t \mid X = x, B = b, , \bar{L}(t\text{-}) = \bar{l}(t\text{-})\}$, the hazard of failure at time t given $X = x$, $B = b$ and $\bar{L}(t\text{-}) = \bar{l}(t\text{-})$ when we intervene to set $\bar{A}(\tau) = \bar{a}$.

Let $\lambda^{*T,\bar{a}}(t \mid \mathcal{F}_{t\text{-}}^*) = \{1 - N^T(t\text{-})\} \times \alpha^{*T,\bar{a}}(t \mid X)$ denote the intensity of the process $\big(N^T(t) : t \geq 0\big)$ with respect to filtration $\mathcal{F}_t^*$ when we intervene to set $\bar{A}(\tau) = \bar{a}$.

The user of our algorithm needs to specify:

- the intensity $\alpha^A\{t \mid X, B, \bar{A}(t\text{-}), \bar{L}(t\text{-})\}$ and the probability mass function $p^A\{a(t) \mid X, B, \bar{A}(t\text{-}), \bar{L}(t\text{-}), dN^A(t) = 1\}$;
- the intensity $\alpha^L\{t \mid X, B, \bar{A}(t\text{-}), \bar{L}(t\text{-})\}$ and the probability mass/density function $p^L\{l(t) \mid X, B, \bar{A}(t\text{-}), \bar{L}(t\text{-}), dN^L(t) = 1\}$;
- a 'risk score' function $h^{\bar{a}}\{t \mid x, b, \bar{l}(t\text{-})\}$ of x, b, $\bar{l}(t\text{-})$ and $\bar{a}(t\text{-})$, which will order individuals with the same value of X but different values of $(B, \bar{L}(t\text{-}))$ according to their potential hazards $\alpha^{T,\bar{a}}\{t \mid X, B, , \bar{L}(t\text{-})\}$.[3]
- the correlation parameter ρ $(-1 \leq \rho \leq 0)$ of a Gaussian copula for the association between the risk score and the failure time;
- the hazard $\alpha^{*T,\bar{a}}(t \mid X)$.

The last of these would be chosen so that the MSM of interest is correctly specified. Dong (2021, page 30) gives the example of $\alpha^{T,\bar{a}}(t \mid X) = \exp\{\theta_0(t) + \theta_1 a(t\text{-})\}$ for some θ_1 and function $\theta_0(t)$ of t. Ryalen et al. (2019, equation (1)) assume $\alpha^{T,\bar{a}}(t \mid X) = b\{X, a(t\text{-})\}^\top \beta(t)$ for some function $b\{X, a(t\text{-})\}$ of baseline covariates X and current treatment $A(t\text{-})$, and some function $\beta(t)$ of t. In their simulation study (Section 4.5), $\beta(t) = \beta$ is a constant.

Let $T^{\bar{a}}$ denote the failure time when we intervene to set $\bar{A}(\tau) = \bar{a}$. For $s < t \leq \tau$, define

$$F_{T^{\bar{a}}}(t \mid X = x, T^{\bar{a}} > s) = P(T^{\bar{a}} \leq t \mid X = x, T^{\bar{a}} > s) = 1 - \int_s^t \exp\{-\alpha^{*T,\bar{a}}(u \mid x)\}\, du.$$

[3]As in Section 3, $h^{\bar{a}}\{t \mid x, b, \bar{l}(t\text{-})\}$ can be replaced by $\nu_t[h^{\bar{a}}\{t \mid x, b, \bar{l}(t\text{-})\}]$, where ν_t is any monotonically increasing function, because only the ranking of $(B, \bar{L}(t\text{-}))$ matters.

In order for this function $F_{T^{\bar{a}}}(t \mid X = x, T^{\bar{a}} > s)$ to be a (conditional) CDF, we need to define its value also for $t > \tau$. Because we administratively censor failure times at time τ, it will not be important exactly how we define $F_{T^{\bar{a}}}(t \mid X = x, T^{\bar{a}} > s)$ for $t > \tau$, provided that it is a continuous increasing function with $\lim_{t\to\infty} F_{T^{\bar{a}}}(t \mid X = x, T^{\bar{a}} > s) = 1$. We can achieve this by, for example, defining $F_{T^{\bar{a}}}(t \mid X = x, T^{\bar{a}} > s)$ for $t > \tau$ as the minimum of $F_{T^{\bar{a}}}(\tau \mid X = x, T^{\bar{a}} > s) + t - \tau$ and 1.

For any fixed x and s, denote the inverse of $F_{T^{\bar{a}}}(t \mid X = x, T^{\bar{a}} > s)$ as $F^{-1}_{T^{\bar{a}}}(u \mid X = x, T^{\bar{a}} > s)$.[4]

Let $H^{\bar{a}}(t) = \lim_{\delta\to 0+} h^{\bar{a}}[t+\delta \mid X, B, \bar{L}\{(t+\delta)\text{-}\}]$.[5] Let $F_{H^{\bar{a}}(t)}(h \mid X = x, T^{\bar{a}} > t) = P\{H^{\bar{a}}(t) \leq h \mid X = x, T^{\bar{a}} > t\}$ denote the CDF of $H^{\bar{a}}(t)$ given $X = x$ and $T^{\bar{a}} > t$.

Our algorithm assumes that the user's choice of risk score function $h^{\bar{a}}\{t \mid x, b, \bar{l}(t\text{-})\}$ satisfies the following condition:

$$\begin{aligned} &\text{If (i) } h^{\bar{a}}\{t \mid x, b_1, \bar{l}_1(t\text{-})\} > h^{\bar{a}}\{t \mid x, b_2, \bar{l}_2(t\text{-})\} \\ &\text{and (ii) } \big(a(u), l_1(u), l_2(u)\big) = \big(a(t\text{-}), l_1(t\text{-}), l_2(t\text{-})\big) \text{ for all } u \text{ such that } t \leq u < s, \\ &\text{then } h^{\bar{a}}\{s \mid x, b_1, \bar{l}_1(s\text{-})\} > h^{\bar{a}}\{s \mid x, b_2, \bar{l}_2(s\text{-})\}. \end{aligned}$$

This condition ensures that if one individual has a higher $H^{\bar{a}}(t)$ value than a second individual with the same value of X, then the first individual continues to have the higher value of $H^{\bar{a}}(t)$ at all later times until one of these two individuals' time-dependent confounder values changes or $a(t)$ changes. The condition would be satisfied if, for example,

$$h^{\bar{a}}\{t \mid x, b, \bar{l}(t\text{-})\} = \lim_{\delta\to 0+} h^{\bar{a}}\big(c(t) + \delta \mid x, b, \bar{l}[\{c(t)+\delta\}\text{-}]\big) \tag{A23}$$

where $c(t)$ denotes the time of the last change in $\bar{l}(t\text{-})$, i.e. $c(t) = \text{argmax}_{s\leq t}\{dN^L(s) = 1\}$, with $c(t) = 0$ if there is no change. Equation (A23) can be interpreted as meaning that the only time that the risk score function $h^{\bar{a}}\{t \mid x, b, \bar{l}(t\text{-})\}$ (for a fixed $\bar{a}$) can change is immediately after there is a change in the value of the confounders.

If the CDF $F_{H^{\bar{a}}(t)}(u \mid X = x, T^{\bar{a}} > t)$ were known,[6] our algorithm would be as follows.

1. Sample X from $p(X)$.

2. Sample B from $p(B \mid X)$.

3. Set $T = \infty$ and sample processes $\big(A(t) : 0 \leq t \leq \tau\big)$ and $\big(L(t) : 0 \leq t \leq \tau\big)$ using intensities $\alpha^A\{t \mid X, B, \bar{A}(t\text{-}), \bar{L}(t\text{-})\}$ and $\alpha^L\{t \mid X, B, \bar{A}(t\text{-}), \bar{L}(t\text{-})\}$, and

[4] That is, $F^{-1}_{T^{\bar{a}}}(u \mid X = x, T^{\bar{a}} > s)$ satisfies $F^{-1}_{T^{\bar{a}}}\{F_{T^{\bar{a}}}(t \mid X = x, T^{\bar{a}} > s) \mid X = x, T^{\bar{a}} > s\} = t$.

[5] This random variable is a function of t, X, B and $\bar{L}(t)$

[6] For example, this CDF is known for the data-generating mechanism used by Ryalen et al. (2019) for a simulation study in their Section 4. In that study: i) there are no covariates X or B; ii) $L(t)$ is binary with $L(0) = 0$ and $L(s) = 1 \Rightarrow L(t) = 1\ \forall t > s$; iii) $\alpha^T\{t \mid \bar{A}(t\text{-}), \bar{L}(t\text{-})\} = \alpha^T\{t \mid A(t\text{-}), L(t\text{-})\}$; and $\alpha^L\{t \mid \bar{A}(t\text{-}), \bar{L}(t\text{-})\} = \alpha^L\{t \mid A(t\text{-})\}$. So, $H^{\bar{a}}(t) = L^{\bar{a}}(t\text{-})$ is binary, $F_{H^{\bar{a}}(t)}(0 \mid T^{\bar{a}} > t) = P\{L^{\bar{a}}(t) = 0 \mid T^{\bar{a}} > t\} = \int_0^t \exp[-\alpha^L\{s \mid a(s\text{-})\}]\, ds$, and $F_{H^{\bar{a}}(t)}(1 \mid T^{\bar{a}} > t) = 1$.

probability mass/density functions $p^A\{a(t) \mid X, B, \bar{A}(t\text{-}), \bar{L}(t\text{-}), dN^A(t) = 1\}$ and $p^L\{l(t) \mid X, B, \bar{A}(t\text{-}), \bar{L}(t\text{-}), dN^L(t) = 1\}$. An example to illustrate how this could be done is given immediately after the end of this algorithm. Denote the sampled value of $\bar{A}(\tau)$ as $\bar{a}$.

4. Set $t = 0$.

5. Let $b = \text{argmin}_{\{s:s>t\}}\{dN^L(s) = 1 \text{ or } dN^A(s) = 1 \text{ or } s = \tau\}$.

6. Calculate $H^{\bar{a}}(t) = \lim_{\delta\to 0+} h^{\bar{a}}[t + \delta \mid X, B, \bar{L}\{(t + \delta)\text{-}\}]$.

7. Calculate $U_H = F_{H^{\bar{a}}(t)}\{H^{\bar{a}}(t) \mid X, T^{\bar{a}} > t\}$ and then $Z_H = \Phi^{-1}(U_H)$. Sample $Z_T \sim \text{Normal}(\rho Z_H, 1 - \rho^2)$ and calculate $U_T = \Phi(Z_T)$.

8. Calculate $T = t + F^{-1}_{T^{\bar{a}}}(U_T \mid X, T^{\bar{a}} > t)$.

9. If $T > b$ and $b < \tau$, set $t = b$ and return to step 5.

10. If $T > \tau$, then T is administratively censored at time τ. Otherwise, if $T \geq \tau$, then T is the failure time and set $A(t)$ and $L(t)$ equal to 'missing' for all $t \geq T$.

In this paragraph, we present a very simple example to illustrate how step 3 could be carried out. Suppose A is binary and L is a single categorical variable with three levels (0, 1 and 2). We shall omit X. In order to allow $A(0)$, the initial value of treatment, to depend on $L(0)$, the intial value of the time-dependent confounders, we shall include $L(0)$ in the vector of baseline variables B. For simplicity, suppose $B = L(0)$ is the only baseline variable. We need to choose a probability mass function for B and a probability mass function $p^A\{a(0) \mid B\}$ for $A(0)$ given B, and sample B and $A(0)$ from these. Now, suppose that for $t > 0$, we choose $\alpha^A\{t \mid B, \bar{A}(t\text{-}), \bar{L}(t\text{-})\} = 0.1 + 0.05L(t\text{-})$ and $\alpha^L\{t \mid B, \bar{A}(t\text{-}), \bar{L}(t\text{-})\} = 0.2 - 0.1A(t\text{-})$ and $p^A\{a(t) \mid B, \bar{A}(t\text{-}), \bar{L}(t\text{-}), dN^A(t) = 1\} = I\{a(t) \neq A(t\text{-})\}$ and $p^L\{l(t) \mid B, \bar{A}(t\text{-}), \bar{L}(t\text{-}), dN^L(t) = 1\} = 0.5I\{l(t) \neq L(t\text{-})\}$. Sample a candidate time to treatment change and a candidate time to confounder change from the exponential$(0.1 + 0.05L(0))$ and exponential$(0.2 - 0.1A(0))$ distributions, respectively. Identify which of these two candidate times is the smaller and call this time t_1. Suppose, for example, that the candidate time to treatment change is the smaller. Set $A(t_1) = 1 - A(0)$ (note this is equivalent to sampling from probability mass function $I\{a(t_1) \neq A(t_1\text{-})\}$). Also, set $A(s) = A(0)$ for all $0 < s < t_1$ and set $L(s) = L(0)$ for all $0 < s \leq t_1$. Now sample candidate time to treatment change and candidate time to confounder change from the exponential$(0.1 + 0.05L(t_1))$ and exponential$(0.2 - 0.1A(t_1))$ distributions, respectively. Identify which of these two candidate times is the smaller and call this time $t_1 + t_2$. Suppose, for example, that the candidate time to confounder change is the smaller. Sample $L(t_1 + t_2)$ from probability mass function $0.5I\{l(t_1 + t_2) \neq L(t_1)\}$. Also, set $A(s) = A(t_1)$ for all $t_1 < s \leq t_2$ and set $L(s) = L(t_1)$ for all $t_1 < s < t_2$. Now sample candidate time to treatment change and candidate time to confounder change from the exponential$(0.1 + 0.05L(t_1 + t_2))$ and exponential$(0.2 - 0.1A(t_1 + t_2))$ distributions,

respectively. Identify which of these two candidate times is the smaller and call this time $t_1 + t_2 + t_3$. Continue in this way until $t_1 + t_2 + \ldots \geq \tau$.

In general, the CDF $F_{H^{\bar{a}}(t)}(u \mid X = x, T^{\bar{a}} > t)$ would be unknown. Therefore, we propose the following algorithm, which, like the extended algorithm in Section 4, uses matches to estimate this CDF at the same time as generating data for a single sampled individual. To avoid complicating this algorithm, we have chosen not to replace matches that fail with copies of randomly chosen matches that have not yet failed. This enables the entire treatment and time-dependent confounder processes for the sampled individual (individual $i = 1$) and the matches (individuals $i = 2, \ldots, m$) to be generated at the very beginning of the algorithm, rather than sequentially during the algorithm, and so reduces the number of steps that we need to write down. However, the algorithm could be modified to replace failing matches with copies of non-failing matches. Such modification could be important if the marginal probability of failure before time τ is high, in which case many of the matches may fail before time τ.

1. Sample X_1 from $p(X)$. Set $X_j = X_1$ for $j = 2, \ldots, m$.
2. For $j = 1, \ldots, m$, sample B_j from $p(B_j \mid X_j)$.
3. Set $T_1 = \infty$ and sample processes $\big(A_1(t) : t \geq 0\big)$ and $\big(L_1(t) : t \geq 0\big)$ using intensities $\alpha^A\{t \mid X_1, B_1, \bar{A}_1(t\text{-}), \bar{L}_1(t\text{-})\}$ and $\alpha^L\{t \mid X_1, B_1, \bar{A}_1(t\text{-}), \bar{L}_1(t\text{-})\}$, and probability mass/density functions $p^A\{a(t) \mid X_1, B_1, \bar{A}_1(t\text{-}), \bar{L}_1(t\text{-}), dN_1^A(t) = 1\}$ and $p^L\{l(t) \mid X_1, B_1, \bar{A}_1(t\text{-}), \bar{L}_1(t\text{-}), dN_1^L(t) = 1\}$. Denote the sampled value of $\bar{A}_1(\tau)$ as $\bar{a}$.
4. For $j = 2, \ldots, m$, set $\bar{A}_j(\tau) = \bar{a}$ and $T_j = \infty$, and sample the process $\big(L_j(t) : t \geq 0\big)$ using intensity $\alpha^L\{t \mid X_j, B_j, \bar{A}_j(t\text{-}), \bar{L}_j(t\text{-})\}$, and probability mass/density function $p^L\{l(t) \mid X_j, B_j, \bar{A}_j(t\text{-}), \bar{L}_j(t\text{-}), dN_j^L(t) = 1\}$.
5. Set $t = 0$.
6. Set $\mathcal{M} = \{j : \ T_j > t\}$ and let $b = \text{argmin}_{\{s:s>t\}}\{dN_j^L(s) = 1$ for some $j \in \mathcal{M},$ or $dN_j^A(s) = 1$ for some $j \in \mathcal{M},$ or $s = \tau\}$.
7. For $j \in \mathcal{M}$, calculate $H_j^{\bar{a}}(t) = \lim_{\delta \to 0+} h^{\bar{a}}[t + \delta \mid X_j, B_j, \bar{L}_j\{(t + \delta)\text{-}\}]$.
8. For $j \in \mathcal{M}$, let R_j denote the rank of $H_j^{\bar{a}}(t)$ among the set $\{H_j^{\bar{a}}(t) : \ j \in \mathcal{M}\}$.
9. For $j \in \mathcal{M}$, sample $W_j \sim \text{Uniform}(0, 1)$, calculate $U_{Hj} = (R_j - W_j)/|\mathcal{M}|$, calculate $Z_{Hj} = \Phi^{-1}(U_{Hj})$, sample $Z_{Tj} \sim \text{Normal}(\rho Z_{Hj}, 1-\rho^2)$, and calculate $U_{Tj} = \Phi(Z_{Tj})$.
10. For $j \in \mathcal{M}$, calculate $T_j = t + F^{-1}_{T^{\bar{a}}}(U_{Tj} \mid X_j, T_j^{\bar{a}} \geq t)$.
11. If $b > \min\{T_j : j \in \mathcal{M}\}$, set $b = \min\{T_j : j \in \mathcal{M}\}$.
12. If $T_1 > b$ and $b < \tau$, set $t = b$ and return to step 6.

13. If $T_1 > \tau$, then T_1 is administratively censored at time τ. Otherwise, if $T_1 \leq \tau$, then T_1 is the failure time of the sampled individual and set $A_1(t)$ and $L_1(t)$ equal to 'missing' for all $t \geq T_1$.

L Discrete risk score

If all the confounders are discrete random variables, then the risk score $H_k^{\bar{a}_k}$, and hence the risk quantile $U_{H_k^{\bar{a}_k}}$, will also be discrete. The copula requires that $U_{H_k^{\bar{a}_k}}$ be conditionally uniformly distributed on the interval $(0, 1)$ given X, which cannot be true if $U_{H_k^{\bar{a}_k}}$ is a discrete variable. To handle this situation, our algorithm can be modified in either of two ways. We shall assume, without loss of generality, that $H_k^{\bar{a}_k}$ takes integer values.

Option 1: Modified Copula

Instead of setting $U_{H_k^{\bar{a}_k}} = F_{H_k^{\bar{a}_k}}(H_k^{\bar{a}_k} \mid X, Y_k^{\bar{a}_{k-1}} = 1)$, generate

$$U_{H_k^{\bar{a}_k}} \sim \text{Uniform}\left(F_{H_k^{\bar{a}_k}}(H_k^{\bar{a}_k} - 1 \mid X, Y_k^{\bar{a}_{k-1}} = 1),\ F_{H_k^{\bar{a}_k}}(H_k^{\bar{a}_k} \mid X, Y_k^{\bar{a}_{k-1}} = 1)\right).$$

This ensures that $U_{H_k^{\bar{a}_k}} \mid X \sim \text{Uniform}(0, 1)$, as required. If we do this, we are now generating $Y_{k+1}^{\bar{a}_k}$ from

$$P(Y_{k+1}^{\bar{a}_k} = 0 \mid X, B, \bar{L}_k) = \int_{U^{\min}_{H_k^{\bar{a}_k}}}^{U^{\max}_{H_k^{\bar{a}_k}}} \Phi\left(\frac{\Phi^{-1}\{g_1(\bar{a}_k, X; \beta)\} - \rho_k \Phi^{-1}(u)}{\sqrt{1 - \rho_k^2}}\right) du$$

where

$$\begin{aligned} U^{\min}_{H_k^{\bar{a}_k}} &= F_{H_k^{\bar{a}_k}}(H_k^{\bar{a}_k} - 1 \mid X, Y_k^{\bar{a}_{k-1}} = 1) \\ U^{\max}_{H_k^{\bar{a}_k}} &= F_{H_k^{\bar{a}_k}}(H_k^{\bar{a}_k} \mid X, Y_k^{\bar{a}_{k-1}} = 1). \end{aligned}$$

Option 2: Odds ratio

When $H_k^{\bar{a}_k}$ is categorical (ordered or unordered) with J levels $(0, \ldots, J - 1)$, we can dispense with the copula and instead specify the $J - 1$ odds ratios:

$$\frac{P(Y_{k+1}^{\bar{a}_k} = 1 \mid X, H_k^{\bar{a}_k} = j)}{P(Y_{k+1}^{\bar{a}_k} = 0 \mid X, H_k^{\bar{a}_k} = j)} \times \frac{P(Y_{k+1}^{\bar{a}_k} = 0 \mid X, H_k^{\bar{a}_k} = 0)}{P(Y_{k+1}^{\bar{a}_k} = 1 \mid X, H_k^{\bar{a}_k} = 0)} \quad (j = 1, \ldots, J - 1)$$

The cell probabilities $P(Y_{k+1}^{\bar{a}_k} = y, H_k^{\bar{a}_k} = j \mid X)$ $(y = 0, 1;\ j = 0, \ldots, J - 1)$ of the $2 \times J$ contingency table for the two categorical variables $Y_{k+1}^{\bar{a}_k}$ and $H_k^{\bar{a}_k}$ can then be calculated from these odds ratios and the marginal probabilities $P(Y_{k+1}^{\bar{a}_k} = y \mid X)$ and $P(H_k^{\bar{a}_k} = j \mid X)$ $(y = 0, 1;\ j = 0, \ldots, J - 1)$. If $J = 1$ (i.e. $H_k^{\bar{a}_k}$ is binary),

this is straightforward. If $J > 1$, it could be done using, for example, the Iterative Proportional Fitting algorithm (see [5] for references).

From these cell probabilities, the conditional probability

$$P(Y_{k+1}^{\bar{a}_k} = 0 \mid X, H_k^{\bar{a}_k}) = \frac{P(Y_{k+1}^{\bar{a}_k} = 0, H_k^{\bar{a}_k} \mid X)}{P(Y_{k+1}^{\bar{a}_k} = 0, H_k^{\bar{a}_k} \mid X) + P(Y_{k+1}^{\bar{a}_k} = 1, H_k^{\bar{a}_k} = j \mid X)}$$

can easily be calculated and then $Y_{k+1}^{\bar{a}_k}$ sampled from this probability.

M MSMs for dynamic treatment regimes

In our article, we considered static treatment regimes, but MSMs are also used for dynamic treatment regimes[14, 2, 4, 6, 7, 16]. For example, Cain et al. (2016)[2] compare two dynamic regimes for HIV-positive patients: starting treatment when patient's HIV RNA exceeds 400 copies/mL, and starting when RNA exceeds 1000 copies/mL. Analyses of this type essentially involve assuming a MSM of the form $P(Y_{k+1}^s = 1 \mid X, Y_k^s = 1) = \text{expit}(\beta_{k0} + \beta_1 X + \beta_{k2} s)$, where X are baseline covariates and Y_k^s is an indicator of survival at visit k when dynamic treatment regime $S = s$ is used ($s = 0, 1$). For example, $S = 1$ and $S = 0$ could mean start treatment when RNA exceeds, respectively, 400 and 1000 copies/mL. The simulation approach Evans and Didelez (2024)[5] describe, and which we have adopted in our article, may not be natural for such regimes, for the following reason. Suppose we omit X from the above MSM and choose $\beta_{00} = 5$, $\beta_{02} = 0$, $\beta_{10} = -5$ and $\beta_{12} = 10$. Then regime $S = 1$ almost guarantees survival to visit 2, whereas $S = 0$ almost guarantees failure between visits 1 and 2. For the two regimes to differ so much, the vast majority of individuals must cross the 400, but not the 1000, copies/mL threshold between visits 1 and 2. However, our approach ostensibly allows the β parameters to be chosen entirely separately from the parameters of the treatment model. For example, we might choose to distribute the treatment start times of patients when assigned to regime $S = 1$ uniformly over the visit times. More research is needed to simulate data for dynamic-regime MSMs.

References

[1] K Aas, C Czado, A Frigessi, and H Bakken. Pair-copula constructions of multiple dependence. *Insurance: Mathematics and Economics*, 44:182–198, 2009.

[2] LE Cain et al. Using observational data to emulate a randomized trial of dynamic treatment-switching strategies: an application to antiretroviral therapy. *International Journal of Epidemiology*, 45:2038–2049, 2016.

[3] Y Dong. *Continuous-time marginal structural models for adverse drug effects in pharmacoepidemiology.* Master of science, Department of Public Health Sciences, University of Toronto, 2021.

[4] L Emilsson, X Garcia-Albeniz, RW Logan, EC Caniglia, M Kalager, and MA Hernan. Examining bias in studies of statin treatment and survival in patients with cancer. *JAMA Oncology*, 4:63–70, 2017.

[5] RJ Evans and V Didelez. Parameterizing and simulating from causal models. *Journal of the Royal Statistical Society Series B*, page in press, 2024.

[6] X Garcia-Albeniz, JM Chan, A Paciorek, RW Logan, SA Kenfield, MR Cooperberg, PR Carroll, and MA Hernan. Immediate versus deferred initiation of androgen deprivation therapy in prostate cancer patients with PSA-only relapse. *Eur J Cancer*, 51:817–824, 2015.

[7] M Hernan. How to estimate the effect of treatment duration on survival outcomes using observational data. *BMJ*, 360:k182, 2018.

[8] MA Hernan, B Brumback, and JM Robins. Marginal structural models to estimate the causal effect of Zidovudine on the survival of HIV-positive men. *Epidemiology*, 11:561–570, 2000.

[9] TS Richardson and JM Robins. Single world intervention graphs (SWIGs): A unification of the counterfactual and graphical approaches to causality. Working Paper Number 128, Center for Statistics and the Social Sciences, University of Washington, 2013.

[10] PC Ryalen, MJ Stensrud, S Fossa, and K Roysland. Causal inference in continuous time: an example on prostate cancer therapy. *Biostatistics*, 21:172–185, 2020.

[11] PC Ryalen, MJ Stensrud, and K Roysland. The additive hazard estimator is consistent for continuous-time marginal structural models. *Lifetime Data Analysis*, 25:1526–1541, 2019.

[12] O Saarela and Z Liu. A flexible parametric approach for estimating continuous-time inverse probability of treatment and censoring weights. *Statistics in Medicine 2016*, 35:4238–4251, 2016.

[13] U Schepsmeier and J Stober. Derivatives and Fisher information of bivariate copulas. *Stat Papers*, 55:525–542, 2014.

[14] JG Young, LE Cain, JM Robins, EJ O'Reilly, and MA Hernan. Comparative effectiveness of dynamic treatment regimes: an application of the parametric g-formula. *Statistics in Biosciences*, 3:119–143, 2011.

[15] JG Young and EJ Tchetgen Tchetgen. Simulation from a known Cox MSM using standard parametric models for the g-formula. *Statistics in Medicine*, 33:1001–1014, 2014.

[16] Y Zhang, M Thamer, J Kaufman, D Cotter, and M Hernan. Comparative effectiveness of two anemia management strategies for complex elderly dialysis patients. *Medical Care*, 52:S132–S139, 2014.

Parameter	True	Unweighted Est	Unweighted SE	Weighted Est	Weighted SE
β_{00}	-2.5	-3.023	0.006	-2.497	0.007
β_{10}	-2.5	-3.185	0.006	-2.489	0.007
β_{20}	-2.5	-3.230	0.005	-2.512	0.007
β_{30}	-2.5	-3.220	0.005	-2.490	0.008
β_{40}	-2.5	-3.216	0.005	-2.498	0.009
β_{50}	-2.5	-3.205	0.006	-2.505	0.010
β_{60}	-2.5	-3.200	0.006	-2.503	0.012
β_{70}	-2.5	-3.182	0.006	-2.479	0.018
β_{80}	-2.5	-3.176	0.007	-2.475	0.016
β_{90}	-2.5	-3.164	0.007	-2.503	0.017
β_1	0.5	0.429	0.003	0.496	0.004
β_2	0.5	0.403	0.005	0.497	0.007
β_3	-1.0	0.255	0.005	-1.002	0.007
β_4	0.0	0.011	0.001	0.001	0.001
β_5	0.0	0.014	0.001	-0.001	0.002
β_6	0.0	0.042	0.001	0.003	0.002

Table A1: Estimates of parameters in MSM obtained with and without inverse probability of treatment weighting, along with estimated SEs, when the marginal probability of failure before time 10 equals 0.5.

Parameter	True	Unweighted Est	Unweighted SE	Weighted Est	Weighted SE
β_{00}	-4.1	-4.845	0.012	-4.106	0.024
β_{10}	-4.1	-5.114	0.013	-4.096	0.022
β_{20}	-4.1	-5.239	0.012	-4.141	0.022
β_{30}	-4.1	-5.273	0.012	-4.101	0.027
β_{40}	-4.1	-5.336	0.011	-4.086	0.040
β_{50}	-4.1	-5.387	0.012	-4.101	0.042
β_{60}	-4.1	-5.417	0.012	-4.077	0.047
β_{70}	-4.1	-5.481	0.013	-4.017	0.099
β_{80}	-4.1	-5.508	0.014	-4.069	0.060
β_{90}	-4.1	-5.579	0.016	-3.908	0.126
β_1	0.5	0.422	0.005	0.533	0.015
β_2	0.5	0.364	0.011	0.523	0.033
β_3	-1.0	0.596	0.012	-1.022	0.024
β_4	0.0	0.001	0.001	-0.007	0.005
β_5	0.0	0.009	0.002	-0.014	0.012
β_6	0.0	0.081	0.002	0.006	0.008

Table A2: Estimates of parameters in MSM obtained with and without inverse probability of treatment weighting, along with estimated SEs, when the marginal probability of failure before time 10 equals 0.1.

Parameter	True	Unweighted Est	Unweighted SE	Weighted Est	Weighted SE
β_{00}	-1.2	-1.572	0.003	-1.197	0.004
β_{10}	-1.2	-1.661	0.004	-1.205	0.004
β_{20}	-1.2	-1.639	0.004	-1.201	0.004
β_{30}	-1.2	-1.593	0.004	-1.204	0.005
β_{40}	-1.2	-1.552	0.004	-1.206	0.007
β_{50}	-1.2	-1.507	0.005	-1.199	0.009
β_{60}	-1.2	-1.477	0.005	-1.212	0.014
β_{70}	-1.2	-1.445	0.006	-1.211	0.017
β_{80}	-1.2	-1.430	0.007	-1.229	0.019
β_{90}	-1.2	-1.394	0.008	-1.184	0.023
β_1	0.5	0.446	0.002	0.500	0.002
β_1	0.5	0.422	0.004	0.495	0.005
β_1	-1.0	-0.012	0.004	-1.002	0.005
β_1	0.0	0.013	0.001	0.001	0.001
β_1	0.0	0.017	0.001	0.004	0.002
β_1	0.0	0.044	0.001	0.003	0.002

Table A3: Estimates of parameters in MSM obtained with and without inverse probability of treatment weighting, along with estimated SEs, when the marginal probability of failure before time 10 equals 0.9.

m	min. I_j	large m
10	0	–
100	10	2000
500	50	10000
1000	100	20000
5000	500	100000

Table A4: The five values of m that were used for this investigation, together with their corresponding thresholds for the minimum number of unique I_j values allowed, and the larger value of m that is used if this threshold is breached.

m	versus $m = 5000$ low	med	high	versus same m low	med	high
10	86.1	63.0	–	90.9	64.3	–
100	93.3	85.3	83.7	92.6	81.0	78.2
500	96.7	92.7	92.0	95.9	90.6	89.4
1000	97.5	94.6	94.0	96.9	93.1	92.3
5000	100.0	100.0	100.0	98.6	96.9	96.6

Table A5: Results for $\rho = -0.9$. For each of the low-risk, medium-risk and high-risk scenarios, the percentage of generated failure times for $n = 100000$ individuals that were the same: a) when $m-1$ matches were used as when 4999 matches were used (columns 2–4); and b) when the same number $m-1$ of matches was used but with two different seeds (columns 5–7). In the high-risk scenario, the data-simulation algorithm sometimes failed when $m = 10$, and so the corresponding entry in the table is "–".

m	versus $m = 5000$ low	med	high	versus same m low	med	high
10	93.0	80.6	–	92.6	78.4	–
100	97.4	92.9	91.0	96.7	90.6	87.9
500	98.8	96.7	95.7	98.5	95.6	94.4
1000	99.1	97.5	96.8	98.9	96.7	95.9
5000	100.0	100.0	100.0	99.5	98.6	98.1

Table A6: Results for $\rho = -0.5$. For each of the low-risk, medium-risk and high-risk scenarios, the percentage of generated failure times for $n = 100000$ individuals that were the same: a) when $m-1$ matches were used as when 4999 matches were used (columns 2–4); and b) when the same number $m-1$ of matches was used but with two different seeds (columns 5–7). In the high-risk scenario, the data-simulation algorithm sometimes failed when $m = 10$, and so the corresponding entry in the table is "–".

	$n = 1000$			$n = 500$			$n = 250$		
	Emp	Sand	Boot	Emp	Sand	Boot	Emp	Sand	Boot
$\delta_{\text{low}}, \rho_{\text{low}}$									
β_1	0.084	0.082	0.083	0.125	0.115	0.117	0.166	0.160	0.168
β_2	0.170	0.167	0.167	0.243	0.234	0.238	0.331	0.326	0.343
β_3	0.251	0.246	0.250	0.367	0.349	0.377	0.542	0.501	1.223
β_4	0.158	0.152	0.154	0.224	0.210	0.216	0.309	0.298	0.320
β_5	0.323	0.310	0.317	0.465	0.439	0.467	0.670	0.626	1.310
$\delta_{\text{high}}, \rho_{\text{low}}$									
β_1	0.157	0.132	0.137	0.198	0.169	0.180	0.253	0.216	0.241
β_2	0.319	0.277	0.288	0.417	0.353	0.379	0.555	0.459	0.533
β_3	0.371	0.326	0.337	0.503	0.422	0.458	0.652	0.560	0.837
β_4	0.232	0.199	0.203	0.290	0.257	0.272	0.384	0.337	0.373
β_5	0.468	0.414	0.428	0.641	0.539	0.581	0.854	0.710	0.969
$\delta_{\text{low}}, \rho_{\text{high}}$									
β_1	0.084	0.084	0.084	0.121	0.118	0.120	0.172	0.165	0.173
β_2	0.169	0.169	0.169	0.242	0.238	0.242	0.351	0.336	0.357
β_3	0.232	0.225	0.224	0.316	0.320	0.328	0.486	0.462	0.786
β_4	0.142	0.139	0.141	0.195	0.196	0.202	0.295	0.282	0.301
β_5	0.292	0.282	0.287	0.422	0.401	0.418	0.641	0.578	0.893
$\delta_{\text{high}}, \rho_{\text{high}}$									
β_1	0.166	0.148	0.151	0.208	0.187	0.199	0.281	0.240	0.275
β_2	0.349	0.304	0.316	0.470	0.396	0.431	0.606	0.522	0.910
β_3	0.352	0.315	0.322	0.481	0.419	0.449	0.630	0.562	0.932
β_4	0.211	0.192	0.196	0.269	0.247	0.261	0.380	0.329	0.368
β_5	0.447	0.397	0.410	0.610	0.525	0.564	0.794	0.704	1.059

Table A7: Simulation study results. Empirical SE of IPTW estimators of $\beta_1, \ldots, \beta_5$ ('Emp'), and mean of estimated SE calculated using the sandwich variance estimator ('Sand') or bootstrap ('Boot'). The maximum Monte Carlo SE associated with these mean estimated SEs is 0.007 and 0.010 when $n = 1000$ and $n = 500$, respectively. When $n = 250$, the maximum Monte Carlo SE is 0.012 for the sandwich method and 0.057 for the bootstrap method.

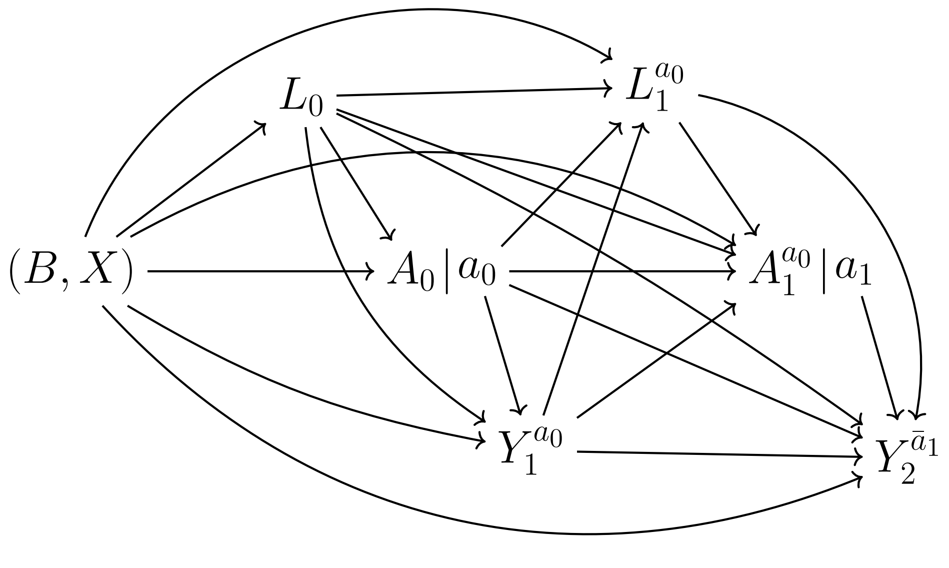

Figure A1: Single World Intervention Graph (SWIG) representing intervention to set $A_0 = a_0$ and $A_1 = a_1$.
